# Spaceflight alters molecular networks linked to diverse human diseases in a single cellular model

**DOI:** 10.1126/sciadv.adw7832

**Published:** 2026-01-02

**Authors:** Wijdan Al-Ahmadi, Rayyanah Barnawi, Edward G. Hitti, Khalid S. A. Khabar

**Affiliations:** ^1^ King Faisal Specialist Hospital and Research Center, Riyadh 11211, Saudi Arabia.; ^2^ Saudi Space Agency, Riyadh 13519, Saudi Arabia.

## Abstract

The International Space Station provides an opportunity to study the impact of spaceflight on gene expression and possible links to human health. Our study investigates global changes in messenger RNA (mRNA) abundance in the THP-1 cell line, a monocyte-macrophage lineage known for plasticity and immune reprogramming features. We identified pathways positively enriched with genes affecting muscle and cardiac contraction, neuronal system, and sensory perception. Available computational models identified links with health issues, including cardiac, neurological, muscular, and renal disorders and alterations in senses. Specific mechanistic networks were identified: retinoid metabolism, cAMP (adenosine 3′,5′-monophosphate)/CREB (cAMP response element–binding protein) signaling, and glutamatergic receptor signaling, which were associated with changes in vision, sleep, and movement, respectively. A considerable reduction is observed in E2F-regulated transcription of G_2_-M and DNA repair genes. A c-myc–regulated mRNA splicing pathway was identified and found commonly down-regulated in other mission datasets. Our results offer a stimulating framework for several health states encountered during spaceflight and can be further used as an accelerated disease and drug discovery model.

## Teaser

RNAomics reveal molecular network changes linked to diverse human diseases during spaceflight using a single cellular model.

## INTRODUCTION

Space travel presents challenges to human health; astronauts display many symptoms and illnesses and are susceptible to infections resulting from apparent changes in several responses, such as immune responses, inflammation, and wound healing (*1*, *2*). Cardiovascular abnormalities have been reported, such as arrhythmias and myocardial insufficiency, in astronauts (*3*, *4*). Space travel can disrupt circadian rhythms and the expression of genes involved in sleep regulation, leading to adverse neurotransmitter interaction and poor sleep quality (*5*). Neuro-ocular syndrome (e.g., visual changes) and neurovestibular symptoms are frequently reported in astronauts (*6*–*8*). Therefore, biomedical space research has gained attention, particularly over recent years (*2*, *9*–*11*). Whole-body investigations and multiomics approaches, including those of cellular models, provide valuable approaches to the understanding of space-related differential gene expression and possible associations with health and disease (*12*).

Molecular, cellular, and physiological changes have been observed during spaceflights, primarily due to exposure to both microgravity and ionizing radiation. Spaceflight affects fluid distribution in the human body, muscle strength, and bone density and causes certain adverse cardiovascular and nervous system events (*1*, *13*). Ionizing radiation, particularly high linear energy transfer radiation such as galactic cosmic rays and solar particle events, poses risks to astronauts’ health (*14*). Although this is of higher magnitude in deep space compared to low Earth orbit, where the International Space Station (ISS) is located, it still presents a series of health problems, particularly with longer missions (*15*). The space environment can alter gene expression, biological processes, and tissue regeneration, potentially contributing to spaceflight illnesses (*1*, *3*, *9*, *10*, *16*, *17*). Adverse biological effects, including those affecting mitochondrial function, DNA damage, immune response, and neurodegenerative processes and oxidative stress, can be influenced by both microgravity and radiation (*14*). Thus, the combined and synergistic effect of microgravity and radiation affects human health in space (*14*, *18*).

Understanding these cellular changes is crucial for developing strategies to mitigate the adverse effects of the space environment and promote astronauts’ health during extended space missions. Moreover, because of the accelerated nature of disease development in the space environment, biological experiments in space can constitute opportunities for studying the progression of chronic diseases on Earth and in a shorter time frame. With laboratory and artificial intelligence models for disease target and drug discovery, spaceflight conditions or simulated microgravity can provide further prospects in the field. In this study, we investigated global gene expression alterations at the cellular level in ISS and their functional and molecular network attributes and applied available machine learning (ML) programs for potential associations with health, disease, and therapy. These detailed analyses were performed with the monocytic-macrophage leukemia line, THP-1, a highly plastic myeloid cell line capable of modeling immune reprogramming and stress-response transcriptional activity.

Although the THP-1 cell line has been extensively used to study monocyte/macrophage functions, such as in inflammatory and immune responses (*19*, *20*), it has the potential to study other biological and disease contexts. This is because monocytes are precursors of macrophages that reside in many parts of the body and are crucial for maintaining tissue-specific homeostasis and functions (*21*–*23*). They are named after anatomical sites such as alveolar macrophages (lungs), Langerhans cells (epidermis), microglia (neuronal system), osteoclasts (bone), and Kupffer cells (liver) (*24*, *25*). Monocytes and macrophages can switch between phenotypes even after differentiation, allowing a high degree of plasticity and heterogeneous gene expression responses (*22*, *26*–*30*). As a matter of fact, chromatin changes and subsequent gene expression changes are well observed during tissue homing (*22*), and both common and distinct transcriptional reprogrammed profiles are found with different microenvironments and stimuli (*26*). Hence, together with the strong effects of the space environment on cellular and molecular processes, our model may present an attractive model for studying disease processes.

Along with ground controls that mimic the same procedures performed aboard the ISS, we used the RNA sequencing (RNA-seq) approach, coupled with rigorous filtering and normalization algorithms. We validated the data with the use of random gene lists of the same size, gene type, and tissue specificity and the use of previous space mission datasets and newly constructed MetaSpace Signature. These insights and analyses led to the identification of diverse affected pathways and bespoke mechanistic networks associating multiple and diverse diseases that match astronauts’ symptoms. All of these, with identifying drugs that have the potential for astronaut health, are achieved with a single cellular model.

## RESULTS

**Fig. 1.**
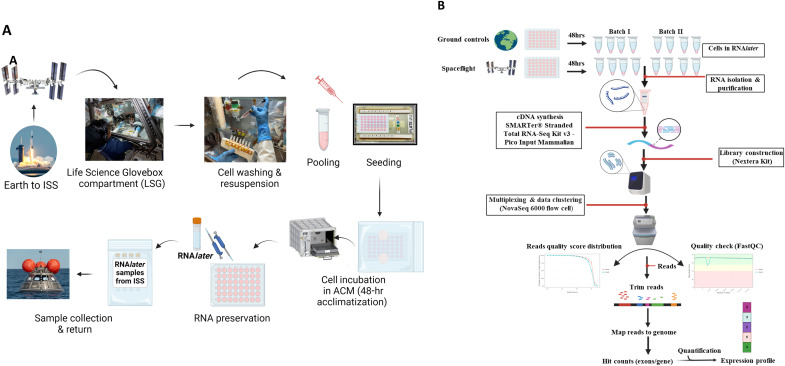
Overview of the mission and experimental workflow for the spacecraft and ground controls. (**A**) Payload and experimental flow at the ISS and ground operations. Details are explained in the Materials and Methods. hr, hour; ACM, atmospheric control module. (**B**) Postspaceflight sample preparation, processing, and RNA-seq. Two batches of samples (different days), each with four replicates from RNA*later* samples, were retrieved from the ISS. Likewise, the same sample formula and procedures were applied to the ground controls that had undergone the same payload handling and conditions (e.g., temperatures). High-quality full-length cDNAs resulting in ribosomal RNA–depleted and low-amplified cDNA libraries were prepared from a total of 16 samples. The final sequencing libraries were multiplexed and clustered on a NovaSeq 6000 flow cell, targeting ~20 million reads per sample. The quality of raw data for each library was examined, then trimmed, mapped to the reference genome, and aligned by bioinformatics to generate hit counts for exons/genes. The sample sequencing statistics for each sample included the size (number of reads and yields in megabase), mean quality score, and % bases >30 (all were within acceptable criteria; see data S6). Moreover, the statistics of mapping are excellent (data S7).

**Fig. 2.**
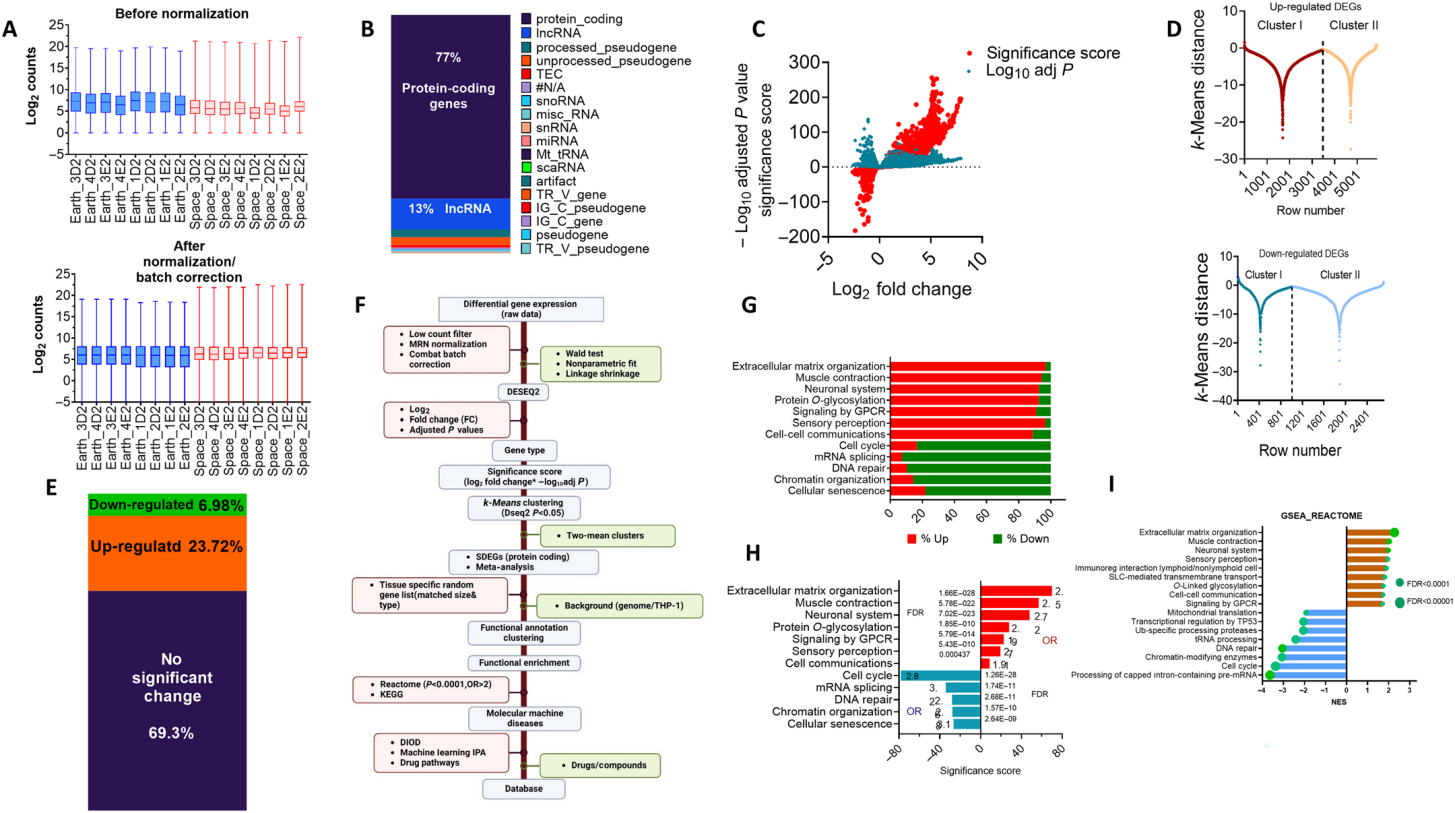
Derivation of significantly expressed genes due to spaceflight exposure using multiple-tier stringent criteria. (**A**) Distribution of read counts from 16 RNA-seq samples representing two batches of experiments at the ISS, each with four replicates (*N* = 8 for space and Earth, respectively). Nearly 20,000 genes for each were obtained after low-count filtering. The read counts were normalized and batch corrected. DEGs were found by using DESeq2 (Wald test, nonparametric fit, and linkage shrinkage). (**B**) Gene types were obtained using Ensembl BioMart and shown as % of all genes. lncRNA, long noncoding RNA. (**C**) Using protein-coding genes, the modified volcano plot shows three parameters: LFC, −log_10_ adjusted *P* value, and SS (= fold enrichment × −log_10_ adjusted *P* values). (**D**) Computational derivation of reliable SS thresholds. SSs for DEGs (adjusted *P* < 0.05) were separated into positive and negative integers corresponding to up-regulated and down-regulated genes. Each group was subjected to the *k*-means clustering algorithm to generate two-mean clusters. The SS thresholds were obtained at the intersection of the two clusters of each group. These statistically significant SDEGs were used for all analyses (data S1). (**E**) Proportion of DEGs that are up-regulated and down-regulated in the spaceflight environment compared to Earth. (**F**) Workflow summarizing the multitier stepwise derivation of SDEGs and determination of the computationally reliable functional enrichment of the SDEGs using background, random lists, and Metalists. (**G**) Reactome analysis was performed on the 4522 SDEGs in the spaceflight-exposed monocytic expressed genome. The data were broadly grouped using the weighted set cover algorithm for redundancy removal; the most significant pathways are shown on the basis of the combined ESS (>10). (**H**) The fold enrichment, FDR, and ESS for the Reactome pathways are shown using the broadest functional categories. (**I**) GSEA for top significant pathways. NESs were obtained after nonredundancy removal. Circles depict FDRs.

The overall methodology for the mission and experimental workflow and the postflight sample processing/sequencing is depicted in Fig. 1 (A and B). RNA sequence data from 16 samples (two batches of independent experiments on two different days at the ISS) were processed, each with four replicates [eight from spaceflight-exposed cells and eight from ground control (Earth)]. We applied stringent criterion parameters to ensure accurate data analysis, including two-tier normalization (Fig. 2A). Principal components analysis (PCA) plots exhibited notable differences between space and Earth groups (fig. S1).

Most detected expressed genes (~19,000, depending on the individual sample) were protein-coding genes (77%; Fig. 2B), a gene type that is the focus of our analysis. To increase the accuracy of differentially expressed genes (DEGs) from DESeq2 output, we applied two-tier criteria. First, we used the gene significance score (SS) method (*31*), which considers both the degree of expression changes and statistical significance. This approach has been thoroughly validated for finding reliable DEGs (*31*). The modified volcano plot shows the log_2_ fold change (LFC), adjusted *P* value (−log_10_), and SS distributions (Fig. 2C and data S1). To find the statistically appropriate threshold for the SS, we used the bimodal *k*-means clustering–based approach (Fig. 2D). This model has been successfully used to segregate low and high signal readings in high-throughput sequence applications (*32*, *33*). The threshold corresponded to the lowest SS of 7.2 equivalent to log_2_ of 2.83-fold × −log_10_ (8.1 × 10^−06^) for up-regulated DEGs and SS of −3.9 equivalent to log_2_ of 0.71 × −log_10_ (1.01 × 10^−08^) for the down-regulated DEGs [these statistically significant DEGs are subsequently designated as spaceflight-affected DEGs (SDEGs)]. There were a total of 31% SDEGs (4522 genes), constituting mostly up-regulated SDEGs (3493 genes), and a lower proportion representing down-regulated SDEGs (*n* = 1028; Fig. 2E and data S1). PCA (fig. S1) indicates further excellent separation between the Earth and space samples (70%), with minimal variance between the samples (~5%).

### Spaceflight-exposed cells exhibit significant differential gene expression patterns

To find functional alterations associated with SDEGs, we applied different bioinformatics and artificial intelligence algorithms (Fig. 2F). We used a background reference human list of all detected expressed genes (14,725 genes) of the protein-coding type. This ensures statistically proper and relevant enrichment results on the basis of the same gene type and tissue specificity (i.e., THP-1 cell line). The use of tissue-selective background is important for accurate identification of overrepresented genes (*34*). Moreover, we generated gene random lists matching the same size of SDEGs (4522 genes), tissue, and gene type (protein-coding regions) generated from all detected genes in THP-1 cells. A total of 4522 SDEGs were analyzed for broad functional categorization using the Reactome database (Fig. 2G). As a control, a random THP-1 gene set was parsed against both the THP-1 background and all protein-coding genes and yielded no Reactome pathway enrichment at a false discovery rate (FDR) <0.05.

We found that the enriched pathways with mainly up-regulated genes (>90%) are extracellular matrix organization, muscle (cardiac) contraction, neuronal system, signaling by heterotrimeric guanine nucleotide–binding protein–coupled receptor, and sensory perception pathways (Fig. 2, G and H). In contrast, the pathways with mostly down-regulated genes (>85%) are mostly linked to selective pressure on DNA processes such as cell cycle, chromatin organization, and DNA repair and RNA splicing (Fig. 2H). We further validated this overrepresentation (ORA)–based analysis with gene set enrichment analysis (GSEA) using also the Reactome database, which largely confirms the same pathways (Fig. 2I). Here, GSEA generates normalized enrichment scores (NESs) with directions of modulation. Additional enriched pathways were observed with an inhibited (negative) direction, including mitochondrial translation (Fig. 2I). The entire details of ORA-based enrichment data are shown in data S2, and GSEA data are shown in data S3. Moreover, table S1 shows a summary of the high-scoring enrichment top-enriched pathways.

### Significantly up-regulated genes during spaceflight relate to cardiac abnormalities

**Fig. 3.**
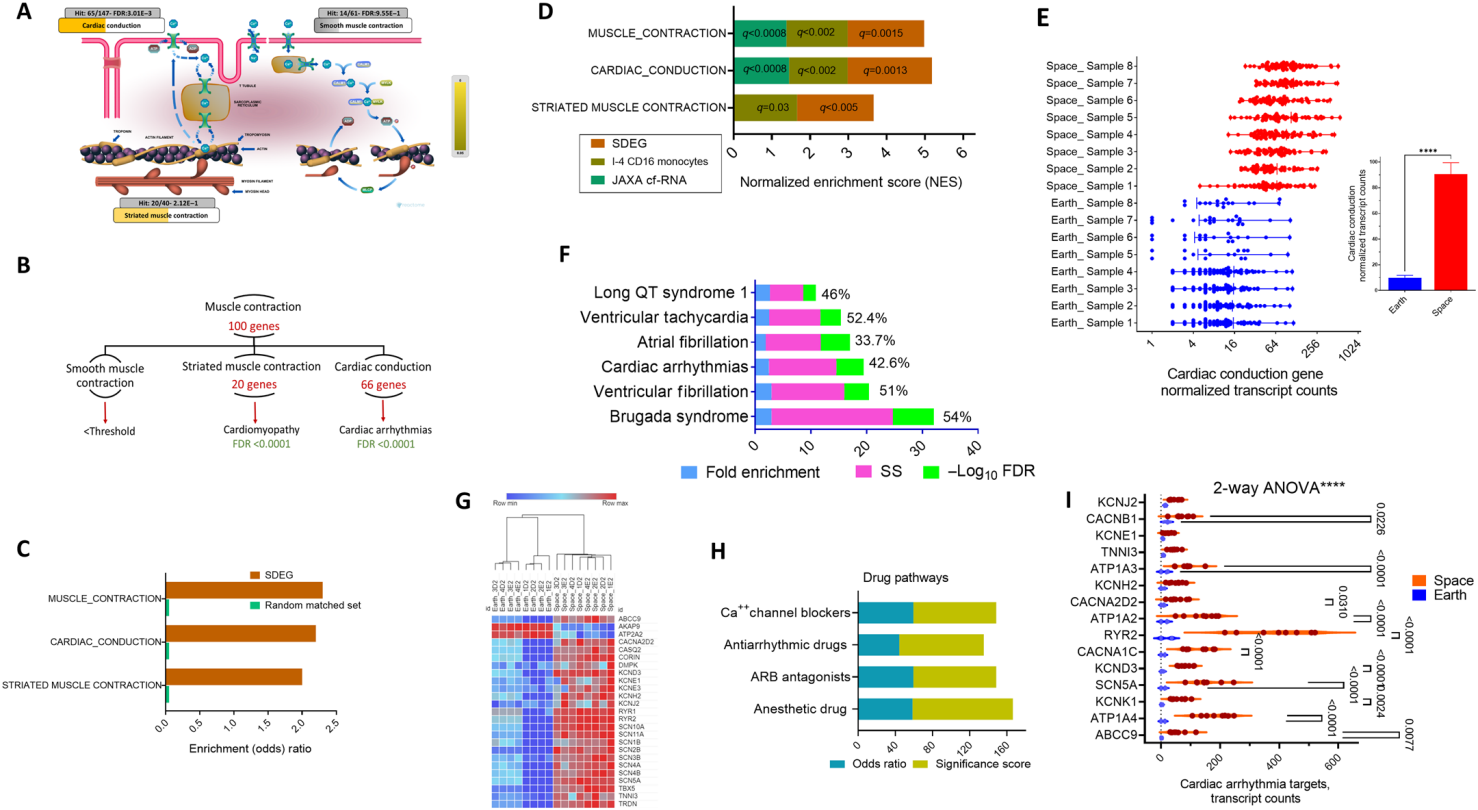
Analysis of muscle/cardiac contraction pathways in the spaceflight-altered expressed genes. (**A**) The event hierarchy of muscle contraction from Reactome analysis was examined, which resulted in three functional groups. FDR was adjusted with the THP-1 background reference and extracted from all DEG enrichment data to avoid dual enrichment. (**B**) The number of genes that comprise each group, mapped disease, and FDR are shown for Reactome hierarchal events. (**C**) Validation of the SDEG pathway enrichment by comparison with the same-size (4521 genes) random sample generated from all detected protein-coding genes in THP-1 (14,746) against the THP-1 background. (**D**) Common pathways detected in SDEG, I-4, and JAXA cf-RNA spaceflight data. Data from SOMA I-4 Multiome_RNA (CD16 monocytes) and fp2 (*R* + 1, *R* + 45, and *R* + 82) versus all preflight as explained in the Materials and Methods. JAXA cf-RNA sequence data were queried with flight versus postflight using the SOMA browser. All calculations were based on GSEA and the overall protein-coding genome to allow comparable analysis. Data are NESs with *q*-values. (**E**) Normalized transcript counts of the up-regulated cardiac conduction SDEGs (*n* = 66). The inset shows the means ± SEM (*n* = 8 per group). ****P < 0.0001, unpaired *t* test with Welch’s correction. (**F**) The top enriched diseases of the 66-gene list, using MOET/ShinyGO disease ontology against the THP-1 background reference, are shown. ESS, % of genes that were mapped to the disease. (**G**) Heatmap of the 24 cardiac arrhythmia SDEGs (LFC). (**H**) Drugs that target cardiac arrhythmia using RGD (human, drug pathways). *P* values were corrected on the basis of the overall SDEG list against the THP-1 background reference list as explained in the Materials and Methods, and the ESS is reported as fold enrichment × −log_10_ FDR. (**I**) Expression patterns of the genes that are antiarrhythmic drug targets using RGD drug pathway data. Two-way ANOVA was performed (****P < 0.0001), and Šídák’s post hoc comparisons between gene rows are shown.

We focused on enriched pathways on the basis of the broad top Reactome categories and their relevance to spaceflight health. We looked first at up-regulated SDEGs (*n* = 3493); we found that the largest enriched group (100 genes) was the muscle contraction pathway [FDR < 2.1 × 10^−15^, 2.7 odds (enrichment) ratio (OR)] (Fig. 3A). This is an important group for space travel; we found that it comprises largely (66%) cardiac contraction pathway genes (2.5 OR, FDR = 1.3 × 10^−11^), and the rest are grouped into striated muscle contraction (Fig. 3, A and B). As a control, these pathways are not enriched with the THP-1 random gene set when compared to our SDEGs (Fig. 3C). For exploration with other publicly available datasets, we looked at these three-muscle contraction groups in Inspiration-4 (I-4)–Space Omics and Medical Atlas (SOMA) Multiome_RNA data. Specifically, we examined the RNA expression I-4 data generated from CD16 monocytes, which closely matched the THP-1 cell line where CD16 is sufficiently expressed (described in detail in the “Cell-type analysis comparison between SDEGs and other space missions’ data analysis” section). To span all the possible variations, we used the datasets of all postflight versus all preflight (fp2 and also fp4) as changes occur in many of the pathways under these conditions (*35*). We found that all the muscle and cardiac conduction groups are significantly enriched in postspaceflight versus preflight astronauts’ blood with our SDEG pathway sets (Fig. 3D). Moreover, we used a different set of publicly available data, the Japanese Space Agency (JAXA) cell-free RNA (cf-RNA) study, which was obtained from six astronauts’ blood in longer ISS flights (120 days) and showed as well that the muscle and cardiac conduction pathways are enriched in the long-duration spaceflight samples (Fig. 3D).

The cardiac conduction gene changes between Earth and space samples (Fig. 3E) were examined, given that space travel may give rise to certain cardiovascular abnormalities. The mean expression of these genes was very low in Earth samples (9.4 normalized transcript counts) but increased by 10-fold (*P* < 0.0001) in the spaceflight-affected cells (90 mean transcript reads; Fig. 3E). Using available ML algorithms for disease association [Multi-Ontology Enrichment Tool (MOET) disease ontology and ML ingenuity pathway analysis (IPA)], there was computational enrichment in many of the cardiac abnormalities related to the overexpression of the cardiac conduction gene group (Fig. 3F), particularly cardiac arrhythmias, including the most common arrhythmia, atrial fibrillation, and also ventricular fibrillation. The largest group belongs to the cardiac arrhythmia group, which comprises 24 genes with their expression increased (6- to 30-fold, *P* < 0.001) in the spaceflight-exposed monocytic cells (Fig. 3G). The most abundantly expressed gene in this group in the spaceflight-affected THP-1 cells was the cardiac ryanodine receptor 2 (*RYR2*), which was 17-fold up-regulated in spaceflight samples. The most induced genes were also *ABCC9* and *KCND3* (27- and 18-fold, *P* < 1 × 10^−20^), which code for sulfonylurea receptor 2 and potassium voltage-gated channel subfamily D member 3, important genes in cardiac arrhythmia. When the other space mission databases were queried through the SOMA browser, at least 50% of these genes existed in the DEG sets of I-4, JAXA cf-RNA, or NASA Twins Study. *RYR2* is also highly inducible in the NASA Twins Study of peripheral blood mononuclear cell samples during ISS flight time spent with an 18-fold increase (*q* = 0.00149).

### Pharmacological inhibitors associated with the reduction of cardiovascular diseases

The cardiac conduction list was further analyzed to identify pharmacological classes for the associated cardiac diseases (Fig. 3H). We used a bioinformatics approach by using MOET (drug pathways). Several drug pathway classes were predicted, including the β-adrenergic receptor selective receptor antagonist (also called β blockers), calcium channel blocking drugs, and even the older class of anesthetic drugs. Nearly a quarter of the cardiac conduction genes in our SDEGs, i.e., 18 genes, are targeted by these drugs. The identity of the genes and their overexpression patterns in spaceflight-exposed cells, whose activities are targeted by these antiarrhythmia drugs, are shown in Fig. 3I. Examples of the specific compounds that are Food and Drug Administration (FDA)–approved drugs are nifedipine, amiodarone, bisoprolol, carvedilol, amlodipine, and others.

### Cardiac myopathies and other muscle-related disease enrichment in spaceflight-induced changes in gene expression

**Fig. 4.**
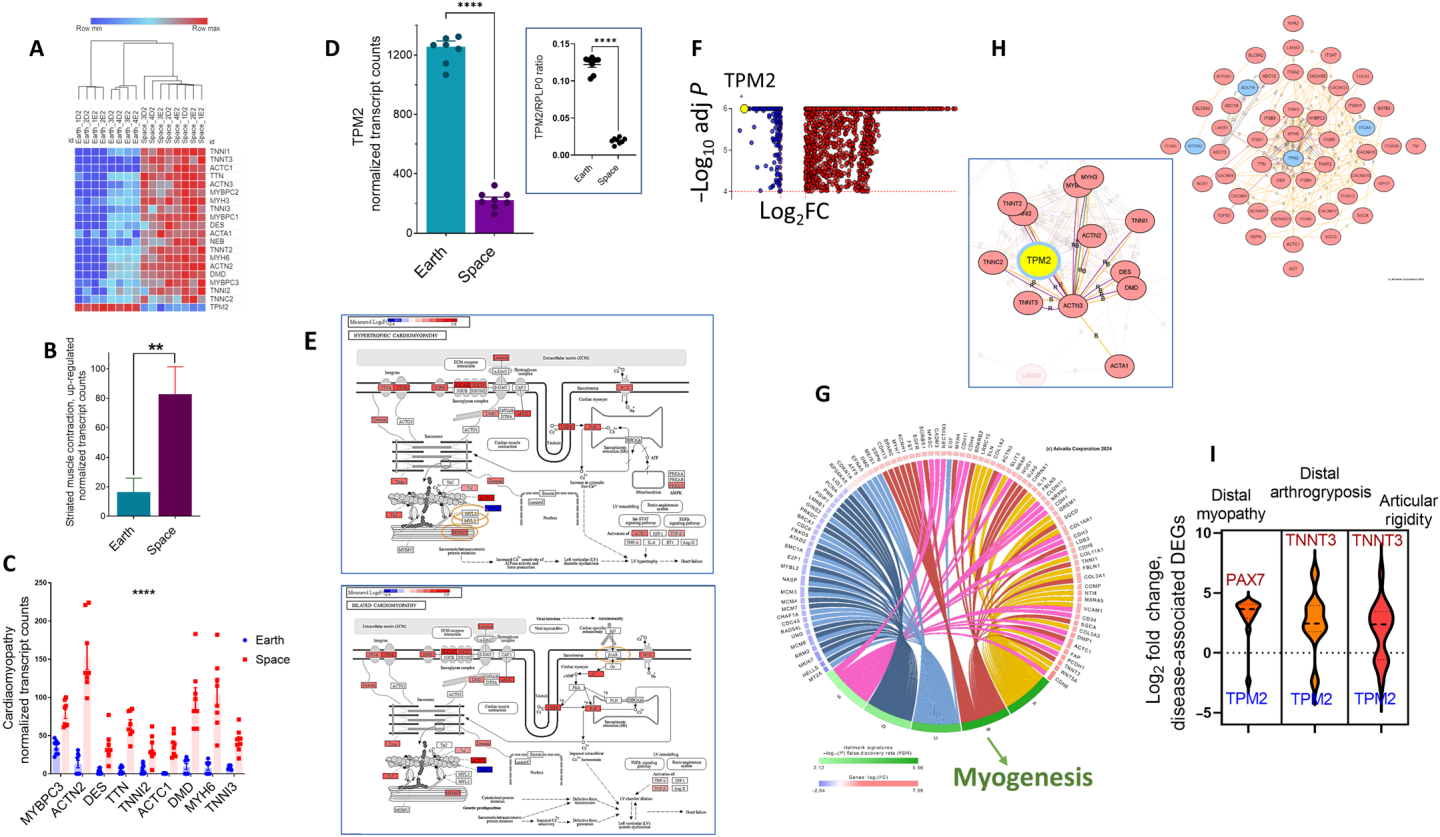
Cardiomyopathy and other myopathy signatures are detected in spaceflight-affected gene expression. (**A**) Heatmap of 20 significantly changed expressed genes in spaceflight-affected monocytic cells versus ground control that belong to the striated muscle contraction gene set. (**B**) Expression plots of the up-regulated DEGs (*n* = 19) associated with the striated muscle contraction in THP-1 monocytes resulting from spaceflight effects. Data are the means ± SEM (*n* = 8 per group). **P = 0.0038 with Student’s *t* test with Welch correction. (**C**) Data are from the individual genes comprising normalized transcript counts (*n* = 8 per group). ****P < 0.0001, two-way ANOVA. (**D**) TPM2 normalized transcript counts presented as individual sample data points or housekeeping *RPLP0* (ribosomal protein lateral stalk subunit P0) normalized ratios. Data are the means ± SEM (*n* = 8 per group). ****P < 0.0001, Student’s *t* test with (inset) or without Welch correction. (**E**) KEGG pathway for the spaceflight-affected SDEGs in hypertrophic and dilated cardiomyopathy. The pathway diagram is overlaid with the computed perturbation of each gene. *TPM2* is the only down-regulated gene product (shown as a blue box); up-regulated genes are shown in red color intensity corresponding to the positive perturbation. The orange-colored circles demonstrate the targets for the indicated drugs. (**F**) Volcano blot shows the SDEGs and *TPM2* (in yellow) as the most significantly down-regulated gene. (**G**) Network analysis was built using the intersection of TPM2-containing pathway members, myogenesis (hallmarks) (**H**), and the cytoskeleton in muscle cells (KEGG pathway). The gatekeeper network showed the effect of TPM2 (yellow) on other interacting genes of the DEGs and was produced with the IPG network analysis algorithm with a high confidence threshold for protein-protein interactions of 0.7. (**I**) Violin plot of differential expression log_2_ of genes comprising the indicated diseases that were identified by an ML program (ML IPA and IPG) at FDR < 0.0001, with the most perturbed genes at the edges.

**Fig. 5.**
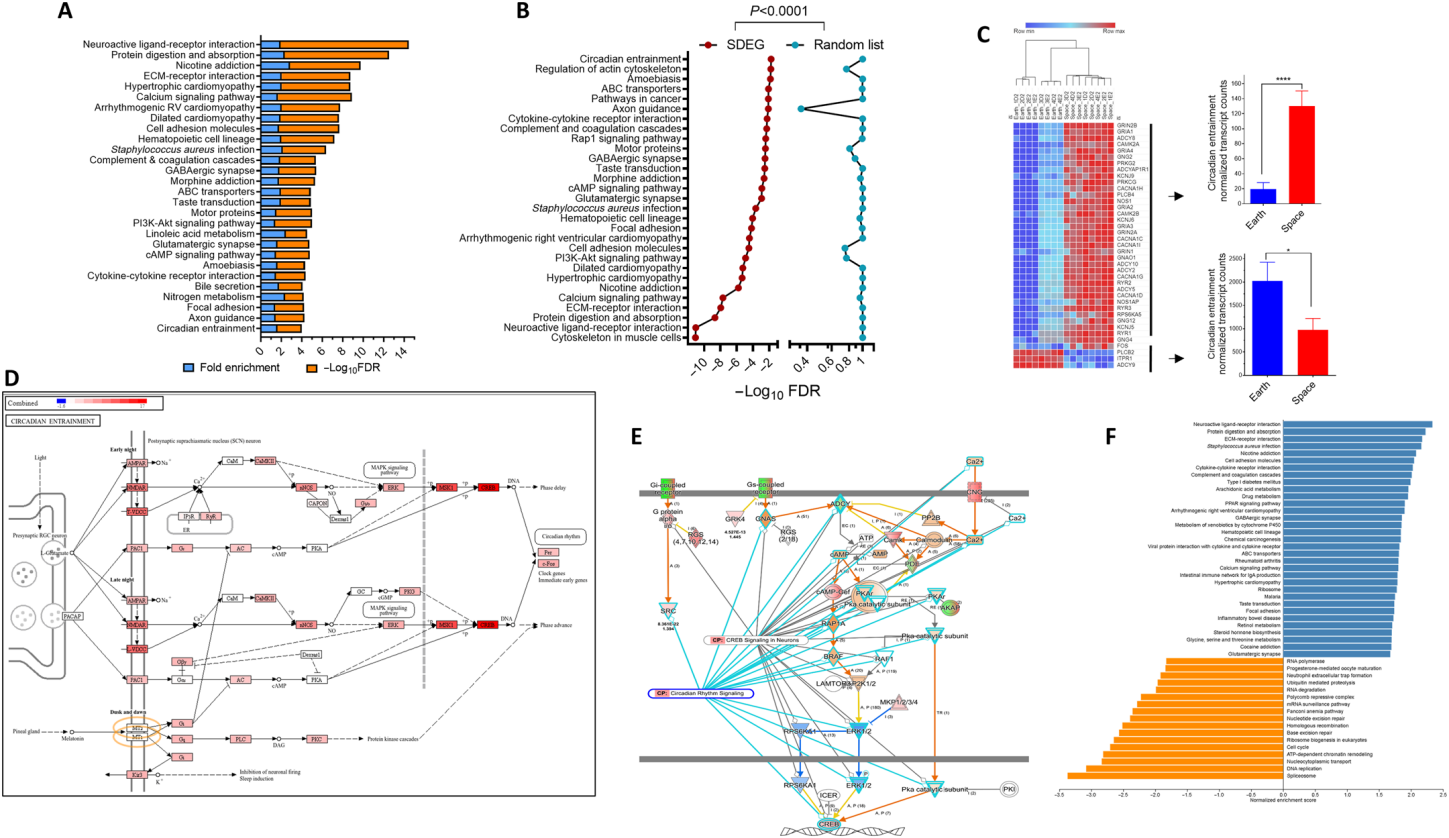
Effect of spaceflight on sleep (circadian entrainment) gene expression. (**A**) KEGG pathway analysis of the entire 4522 SDEGs in the spaceflight-affected THP-1 monocytes. The plot graph shows the fold enrichment and FDR ranked from the top by ESS (fold enrichment × −log_10_ FDR). (**B**) Two datasets (SDEGs versus THP-1–derived random list; 4500 genes each) were analyzed by IPG pathway meta-analysis. FDR was used for *P* value correction. (**C**) Heatmap of 39 significantly changed expressed genes in spaceflight-affected monocytic cells versus ground control that belong to the circadian entrainment pathway. Right panel: means ± SEM (*n* = 8 per group) of gene expression data between spaceflight and Earth control groups for the up-regulated and down-regulated circadian DEGs. ****P < 0.0001, *P < 0.05 and Student’s *t* test. (**D**) Circadian entrainment pathway with the possible role of melatonin affecting the DEGs. The analysis was performed using KEGG network analysis coupled with combined effects of log_2_ expression and downstream effect using IPG algorithms. (**E**) A network was built by overlaying (gray lines) and integrating the action of cAMP pathway and CREB signaling in neurons (shown in squared boxes) with circadian entrainment using IPA network modeling. Blue-highlighted lines show interactions with circadian rhythm signaling. (**F**) GSEA output showing NES values with + or − depicting the direction of pathway modulation being positively enriched (blue bars) or negatively enriched (orange color), respectively.

There were 20 SDEGs in the striated muscle contraction group (Fig. 3B); all were up-regulated during spaceflight, except tropomyosin 2 (*TPM2*), which was down-regulated (Fig. 4, A and D). The overexpression of the SDEGs was fivefold when compared to Earth control (normalized transcript counts at Earth, 16.4 ± 9.4 versus 82.8 ± 18.7 in space; *P* < 0.001) (Fig. 4B). Using disease ontology mapping, nearly half of these genes are significantly related to the disease of hypertrophic cardiomyopathy and dilated cardiomyopathy [the FDR of Kyoto Encyclopedia of Genes and Genomes (KEGG) cardiomyopathy with all DEGs was 4.12 × 10^−06^] and up-regulated (FDR < 0.0001) (Fig. 3B), with an average of 7.4-fold increase (*P* < 0.0001; Fig. 4C). The dilated cardiomyopathy pathway is also enriched (1.94, *q* = 0.000137) in the DEG set of I-4 mission, postflight versus preflight (CD16 monocyte and fp2 data). Dissecting molecular pathways using KEGG (Fig. 5A) demonstrated two prominent signaling pathways that underlie cardiac conduction genes, namely, calcium signaling pathways and adrenergic signaling in cardiomyocytes comprising 35% of the cardiac conduction gene group. The mechanistic pathway diagram showed the target receptors for the drugs: bisoprolol and carvedilol targeting the ADRB1 (β1-adrenergic receptor) and afecamtin and mavacamtin targeting cardiac myosins MYH3 (myosin heavy chain 3) and MYH7 (Fig. 4E).

*TPM2* was one of the most significantly down-regulated genes (80% reduction, adjusted *P* < 4.2^−77^ in the SDEGs) (Fig. 4F). A common gene list was from the two groups (myogenesis hallmarks; Fig. 4G) and the cytoskeleton in muscle cells (KEGG pathway; fig. S2). Network analysis was built from this common set (Fig. 4H). Gatekeeper network building demonstrates that TPM2 is a key gene product with other SDEG-interacting genes in the muscle differentiation processes (Fig. 4H). Using ML disease mapping, there were three further significant enrichments in TPM2-related diseases in the monocytic SDEG list, resulting from spaceflight effects, namely, distal arthrogryposis, congenital myopathy, and articular rigidity (Fig. 4I, FDR < 0.001). TPM2 was the most commonly negatively perturbed (down-regulated) gene product in these three diseases, whereas *TNNT3* (troponin T3, fast skeletal type) and *BAX7* (BCL2-associated X protein 7) are the most abundantly overexpressed (Fig. 4I). TNNT3, a skeletal muscle troponin, was perturbed (up-regulated) compared to the troponin TNNT1, a cardiac muscle troponin. It should be noted that muscle changes during both short and long flights are well-described observations in astronauts [reviewed in (*36*)].

### Molecular pathway analysis of neuronal system genes in SDEGs indicates changes in sleep-light interaction patterns and potential reversal by melatonin action

One of the most affected broad pathways in the SDEGs in THP-1 monocytic cellular gene expression is the neuronal system (Fig. 2, G and H). KEGG pathway analysis was performed to find neuronal system abnormalities that can be traced to health abnormalities during space travel (Fig. 5A). Given that sleep disturbances are known in astronauts during space travel, we noted that the circadian entrainment gene group was enriched in the SDEGs (OR = 1.62 fold, FDR = 0.004). The use of the THP-1 random gene list in KEGG analysis showed no enrichment for circadian entrainment at FDR < 0.05 (Fig. 5B). Circadian rhythm is a prominent pathway enriched in DEGs of other mission/multiomics analysis (*37*), and alterations were found even in multiple organ tissues, which confirms our results.

Most of the genes in the circadian gene group (85%) were overexpressed compared to down-regulated SDEGs in the spaceflight-affected THP-1 monocytes (Fig. 5C). This 40-gene set comprises 50% of all the pathway genes in the genome, where THP-1 that expressed random control lists yielded 0%. Network mapping and drug interactions show that melatonin and its approved drug analogs, agomelatine, ramelteon, and tasimelteon, target this pathway (Fig. 5D). Melatonin is known to inhibit adenylyl cyclase, leading to sleep induction; the adenylyl cyclase genes in the SDEGs, i.e., *ADCY2* (adenylate cyclase 2), *ADCY8*, and *ADCY10*, are overexpressed and possibly normalized by melatonin or its drug analogs in astronauts’ health context (Fig. 5D). Two mechanisms involved in the circadian entrainment are overrepresented in the SDEG set: cAMP (adenosine 3′,5′-monophosphate) response element–binding protein (CREB) signaling in neurons and cAMP signaling. The circadian entrainment pathway culminates in CREB inactivation through inhibition of cAMP production by adenylyl cyclases, as shown mechanistically in the diagram (Fig. 5E). GSEA using KEGG was performed for further validation of the molecular pathways (Fig. 5F and data S3). Most of the pathways that were demonstrated to be up-regulated were also found with GSEA with positive NES values (FDR < 0.005), including those of the sensory and neuronal systems. Enriched pathways with negative NES values as a result of the spaceflight impact on the cells were also demonstrated with ORA-based analysis (data S2).

### Analysis of changes in the sensory system gene expression in THP-1 cells as a result of spaceflight

**Fig. 6.**
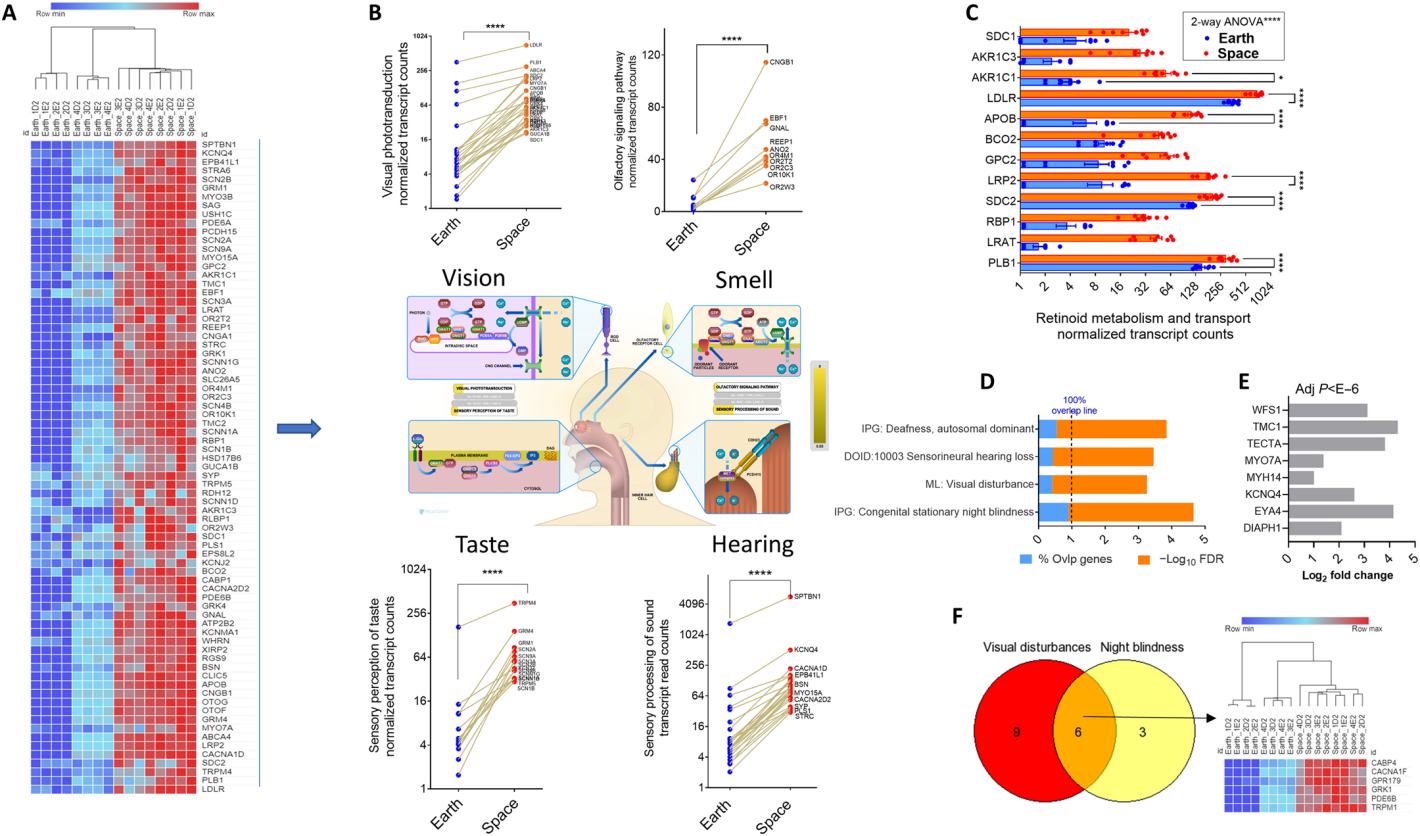
Alterations of sensory system gene expression: Taste, smell, hearing, and vision are found in spaceflight-affected gene expression. (**A**) The sensory perception group (from Fig. 2, G and H) comprising mostly 75 up-regulated genes was plotted as a heatmap (normalized log_2_ ratios) from eight space samples and eight Earth controls, representing two batches each with four replicates. Two-way clustering (single linkage, with Euclidean distance algorithm) was performed, and the heatmap is visualized with Morpheus. (**B**) The sensory perception SDEGs were divided into subpathways using Reactome hierarchy with FDR shown in insets. Individual up-regulated genes as normalized transcript counts in each subpathway are shown with connecting lines between the space and Earth control. The means ± SEM (*n* = 8 per group) for each subpathway gene group are shown in each plot. ****P < 0.0001, two-way ANOVA. (**C**) Retinoid metabolism and transport represent a Reactome subpathway in the visual transduction group. Data are normalized transcript counts for each DEG (*n* = 8 per group). The means ± SEM are shown. Two-way ANOVA was performed with Šídák’s multiple comparisons test. *P < 0.05, ****P < 0.0001. (**D**) ML disease mapping showing the most significantly represented diseases as a result of the sensory perception DEGs (75 genes). The analysis was performed using IPA ML and IPG programs. Data are % of the disease-gene pathway and FDR (as −log_10_). (**E**) LFC and adjusted *P* values for the eight common genes between the hearing loss and autosomal dominant deafness disease pathways. (**F**) Venn diagram showing the six common genes between the two annotated diseases related to vision disturbances and congenital autosomal blindness. Heatmap visualization of the changes in gene expression of the five common genes.

Alterations in sensory inputs such as taste, smell, hearing, distance, and vision are well-documented symptoms in short- and long-duration spaceflights (*25*, *38*). The sensory perception group, as a result of the broad Reactome analysis (Fig. 2, G and H), comprises one of the top significantly enriched pathways (75 genes, FDR < 10^−10^). Nearly all (>96% of the genes) are up-regulated in the spaceflight-exposed THP-1 monocytic cells compared to Earth controls (Fig. 6A). To identify the relationships between these alterations in senses that happen in astronauts and in our gene expression data, the sensory perception group was further subdivided into specific sense type–associated subgroups using Reactome mapping analysis (Fig. 6B, middle diagram). The following pathways were found: sensory perception of sound, sensory perception of taste, olfactory (smell) pathway, and visual transduction (Fig. 6B). We found that in other multiomics, when compared to our study, certain sensory pathway-gene expression alterations are also noted, particularly in the olfactory sensory system (*37*).

In the visual transduction group, 12 genes belong to vitamin A metabolism, retinoid metabolism, and transport pathway, which were significantly altered (up-regulated) in space samples (Fig. 6C). Changes in the expression of *PLB1*, one of the highly expressed and significantly induced genes in our SDEGs, can affect the retinol pathway (*39*). Health issues related to the 75 sensory perception genes are of importance in space medicine because some of the observed symptoms have been examined using ML algorithms (Fig. 6D). These included symptoms related to vision and hearing. Hearing abnormalities were identified by two different ML programs, namely hearing loss, in which 26 SDEGs of the 60-gene disease pathway were annotated (OR = 2.1, FDR < 0.001; Fig. 6D), and autosomal dominant deafness, in which 27 SDEGs of the 50-gene disease pathway were annotated (FDR < 0.001) with eight shared up-regulated genes (Fig. 6E). Visual disturbance (15 SDEGs, which account for 42% of the disease pathway) and night blindness disease (nine SDEGs accounting for the majority of the disease pathway) were annotated to six common genes (Fig. 6F). Although the mutational variants of these genes are causes of hearing loss or vision, the general disturbance in the expression of these genes in spaceflight may still affect hearing and vision in astronauts.

### The glutamatergic receptor signaling pathway is prominent in the spaceflight-affected gene expression with network association with neurological diseases

**Fig. 7.**
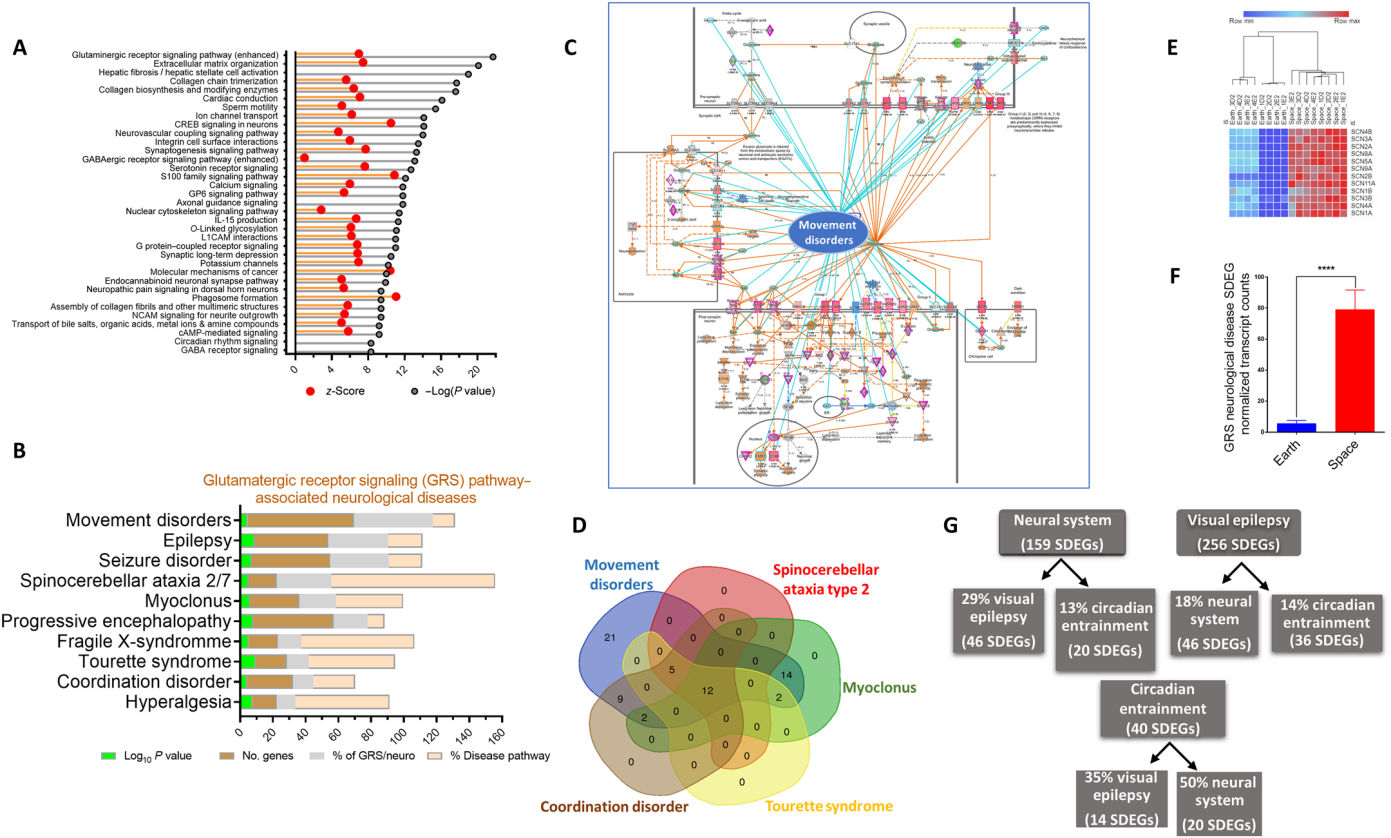
Nervous system diseases and GRS pathway relationships to spaceflight-altered cellular gene expression. (**A**) SDEGs in THP-1 monocytic cells were analyzed for IPA canonical pathways. The *P* value is for Fisher’s exact test for overlap, and the *z*-score determines the degree of activation/inhibition. (**B**) The GRS pathway (134 genes) retrieved from IPA canonical pathway analysis was further examined using the IPA disease function module. The top 10 diseases based on the THP-1 background, corrected *P* value, and Fisher’s exact *t* test are shown. (**C**) GRS pathway network with an overlay of the highest gene number–containing category (movement disorders, 65 genes). Light blue lines show the overplayed edges between the pathway and the disease, while red and blue colors indicate increased or decreased measurements/activities, respectively. (**D**) Venn diagram showing the common genes among the different classified diseases in the movement disorders list. (**E**) Heatmap and (**F**) means ± SEM (*n* = 8 per group) of expression levels of the 12 common movement-related disease genes that change (all were up-regulated) between spaceflight and Earth samples (****P < 0.0001, Mann-Whitney *t* test). (**G**) Enriched network of circadian-visual epilepsy. Edges show % overlapped genes between two nodes.

One of the most affected pathways in the spaceflight-affected cells, according to the KEGG pathway, is neuroactive ligand-receptor interactions (Fig. 5A), particularly glutamatergic and γ-aminobutyric acid receptor signaling (Fig. 7A). Both pathways were enriched in SDEGs as compared to Earth controls (FDR < 10^−21^), also validated with a random THP-1 list (Fig. 5B). When closely examined, these pathways are important in many neurological diseases of relevance to spaceflight. By using ML IPA analysis, we found that a number of these neurological diseases are linked to the alterations in the glutamatergic receptor signaling (GRS) pathway in the spaceflight-subjected THP-1 monocytic cells. There were 135 SDEGs in this pathway, of which the majority (94%) are associated with neurological diseases. The top 10 diseases/conditions associated with this pathway are shown (Fig. 7B). The most affected disease category that involves the GRS pathway is movement disorders (65 genes), which comprises nearly half of GRS-neurological diseases-SDEGs (*P* < −4.6 log_10_; Fig. 7, B and C). Movement disorders are notable in astronauts, particularly in long-duration spaceflights (*40*, *41*). Related movement disorders identified by the algorithm include myoclonus, spinocerebellar ataxia types 2 and 7, coordination disorders, fragile X-linked syndrome, and Tourette syndrome (Fig. 7B)—it should be noted that this does not mean that these diseases are displayed in the astronauts, but rather, they may develop certain movement symptoms that are found in these diseases. There were 12 overlapping genes (Fig. 7D), and they were all overexpressed and shown as a heatmap (Fig. 7E) or as the weighted average of expression values of SDEGs (spaceflight versus Earth control) (Fig. 7F).

Another notable condition that is enriched in the neuronal system is visual epilepsy (46 SDEGs), which comprises 29% of the neuronal system genes, while visual epilepsy as one disease group (256 SDEGs; Fig. 7F) comprises 32.4% of the genome-wide visual epilepsy pathway (791 genes). Circadian entrainment is connected to both the neuronal system and visual epilepsy pathways, which account for 50 and 35% overlap, respectively (Fig. 7G).

### Cell-type analysis comparison between SDEGs and other space missions’ data analysis

**Fig. 8.**
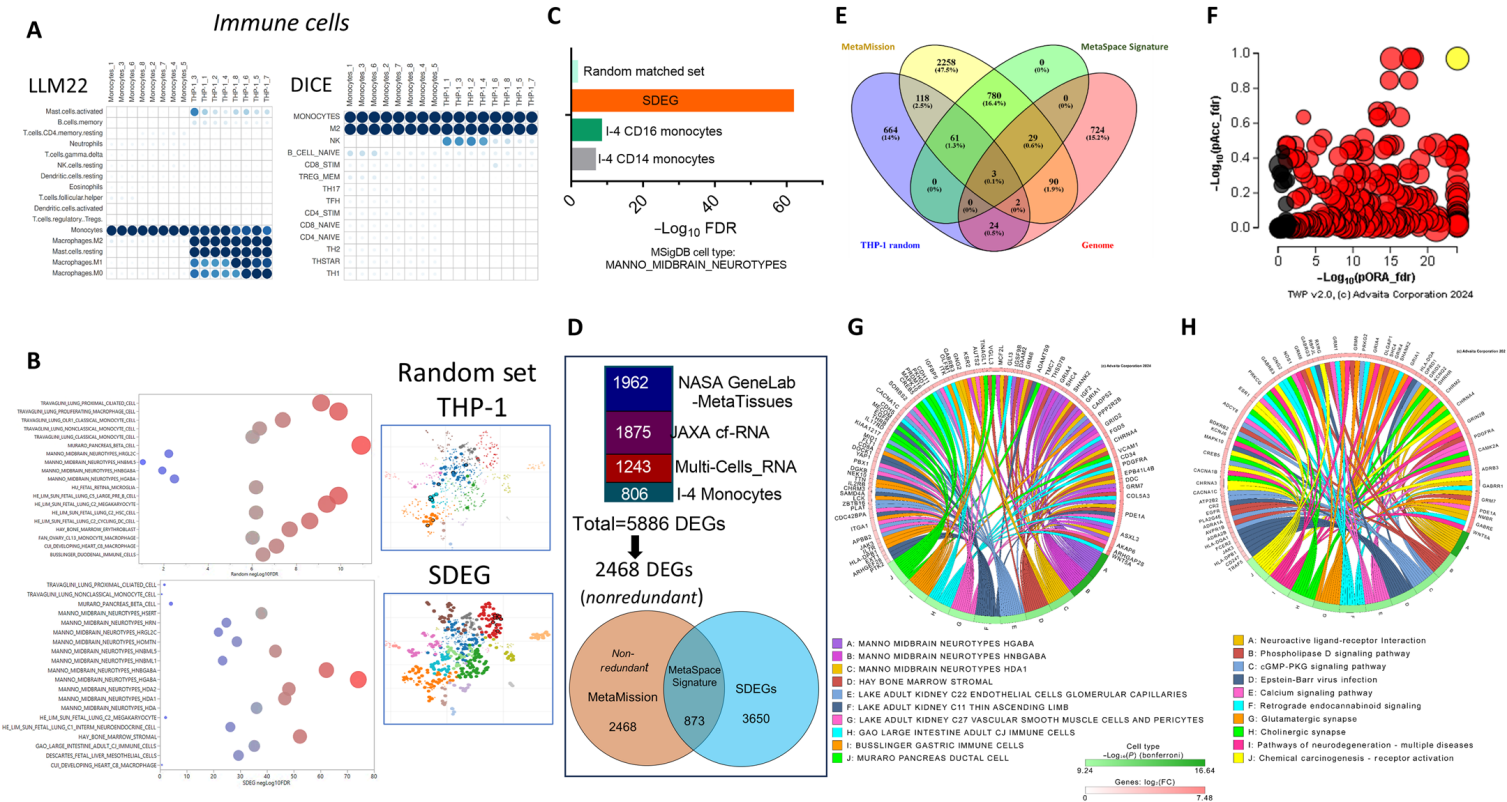
Cell-type mapping and cross-validation of SDEGs against combined and common meta-RNAomics. (**A**) Cell-type mapping profiles for THP-1 Earth control (*n* = 8) samples and normal monocytes (*n* = 8, public dataset; Materials and Methods) using RNA-seq data. Two reference databases, LLM22 (left panel) and DICE (right panel), were used. Dot plot intensity corresponds to significance (BigOmics Analytics). (**B**) Cell type–based gene expression in the space-affected THP-1 cells. The 4522 random gene lists from THP-1 or our SDEG list were used for UMAP analysis. Plots showed 22 clusters that were identified by the UMAP analysis derived from MSigDB with cell-type legends on the left; the *x* axis is −log_10_ FDR. (**C**) I-4 database subsets, as indicated, were queried using the SOMA browser for cell types for comparison using MSigDB cell types. (**D**) Derivation of the combined MetaMission gene set (3340 nonredundant genes) from 5887 DEGs obtained from the indicated databases as explained in the Materials and Methods. The MetaSpace Signature (873 genes) was obtained as the overlap set between the generated MetaMission dataset and our SDEGs. (**E**) Venn diagram demonstrating the overlapping genes among the combined MetaMission and MetaSpace Signature against each of random sets of THP-1 or genome. (**F**) Pathway perturbation versus ORA. Pathways are plotted in terms of the two types of evidence computed by IPG: ORA (pORA, *x* axis) and the total pathway accumulation (pAcc, *y* axis). Each pathway is represented by a circle, with significant pathways shown in red and nonsignificant ones in black, and the size of each dot is proportional to the size of the pathway. Enrichment of (**G**) cell types and (**H**) top pathways summarized by chord analysis. Chord analysis connects the pathway with the most significantly changed genes. FDR values are shown as negative log_10_ for both pathway enrichments (depicted in the color legend). LFCs are also depicted as color legends.

We examined the characteristics of THP-1 monocytic cells as compared to normal monocytes using basal expression data. Cell mapping profiling infers the type of cells by using a computational deconvolution method and expression databases of immune cells: DICE [Database of Immune Cell eQTLs (expression quantitative trait loci), Expression, and Epigenomics] and the single-cell sequencing Large Language Model (LM22) database. We found that the THP-1 cell type maps to largely normal monocytes and macrophages at the transcriptome level (Fig. 8A). A comparison of GSEA hallmark enrichments demonstrated that THP-1 shares an immune/inflammatory response and other related processes with normal monocytes, albeit with reduced expression. However, this still does not mean that THP-1, of cancerous origin, is fully representative of normal monocytes; expectedly, higher abundance with cell cycle enrichment pathways is noted with THP-1 (fig. S3).

Our analysis indicates that gene expression in the THP-1 cell line is strongly influenced by spaceflight conditions, likely relating to both features of monocyte-macrophage plasticity and spaceflight conditions (e.g., microgravity and radiation). Thus, for further validation of our data, we investigated the changes in cell type–specific gene expression and compared the analysis with other space mission datasets. We compared our SDEG dataset and the random gene dataset of the THP-1 background against the protein-coding genome. Uniform manifold approximation and projection (UMAP) analysis clearly shows the redistribution of the cell type–specific gene expression patterns (Fig. 8B and data S4). THP-1 (random gene list) was largely mapped to monocytes and macrophage-type cells as expected (Fig. 8B, upper panel). As a negative control, when the THP-1 random gene list was parsed against the 14,725 THP-1 background gene list, no statistical significance enrichment was obtained, confirming further the power of using random gene lists. We noted neurotype gene expression patterns in SDEGs (47% of the cell-type gene pathway, FDR < 10^−36^; Fig. 8B, lower panel) compared to the random THP-1 list (22.6% of the cell-type gene pathway, FDR = 0.33). The spaceflight-induced changes in cell-type gene expression patterns corresponded to the affected neuronal system pathways. When compared to I-4 CD14/16 monocytes using the SOMA browser, there was similar and statistically significant enrichment, as shown with clusters of neurotype cells (Fig. 8C).

### Cross-comparison and validation of SDEGs against combined and common meta-RNAomics

For further validation of not only the cell-type gene expression but also enriched pathways, we adopt a two-step approach for the comparison of our SDEGs with other spaceflight omics data. The following datasets were used: (i) The NASA GeneLab Meta-Tissue set represents human homologs of DEGs (*n* = 1962) from the mouse RNA-seq data of 27 datasets encompassing 10 different mouse tissues (adrenal glands, colon, eye, kidney, liver, lung, skin, muscle, spleen, and thymus) and obtained from the I-4 study. (ii) A total of 1875 JAXA cf-RNA study DEGs were processed from the RNA-seq data using the plasma of six astronauts’ blood and obtained from NASA GeneLab. (iii) The multicell RNA set is combined from DEGs of the datasets of activated T cells, human endothelial cells, human fibroblasts, and follicular hair. (iv) A total of 806 DEGs were obtained from the I-4 omics study using RNA-seq data of CD14+ and CD16+ monocytes. A combined gene list of these four dataset groups is generated and comprises 5887 DEGs, leading to 56.7% of nonredundant (3340) protein-coding DEGs called here MetaMission (Fig. 8D). The second metaset comprises the overlapped DEGs between MetaMission and our 4522 SDEGs, resulting in 873 genes, called here the MetaSpace Signature (Fig. 8D). A proportion of the MetaSpace Signature (26%) in the MetaMission set was found to be significantly higher (*P* < 0.00001, chi-square test) than the proportion of THP-1 and genome random lists of the same size (5.5 and 3.7%, respectively) (Fig. 8E). We found that there is an appreciable proportion of common functional pathways between the MetaSpace and our SDEG signatures (fig. S4). A Venn diagram was used to demonstrate the overlap between each of the common genes (MetaSignature SDEG), the THP-1 background, and all protein-coding genes—each of a comparable size of 873 genes–against the combined MetaMission gene set (Fig. 8E).

The common 873-gene MetaSpace Signature was examined for pathway enrichment by parsing the lists against the protein-coding genome background, taking into account the two parameters of representation and perturbation score combination (Fig. 8F). The MetaSpace Signature set also confirmed the neurotype cell-type enrichment (Fig. 8G) and several KEGG pathway enrichment (Fig. 8H), including the relevant processes that underlie the neurological and cardiovascular diseases analyzed above such as neuroactive ligand-receptor interactions and the calcium signaling pathway, in addition to the cGMP (guanosine 3′,5′-monophosphate)-PKG (cGMP-dependent protein kinase) signaling pathway.

### Many down-regulated genes are controlled by E2F family transcription factors and cell cycle

**Fig. 9.**
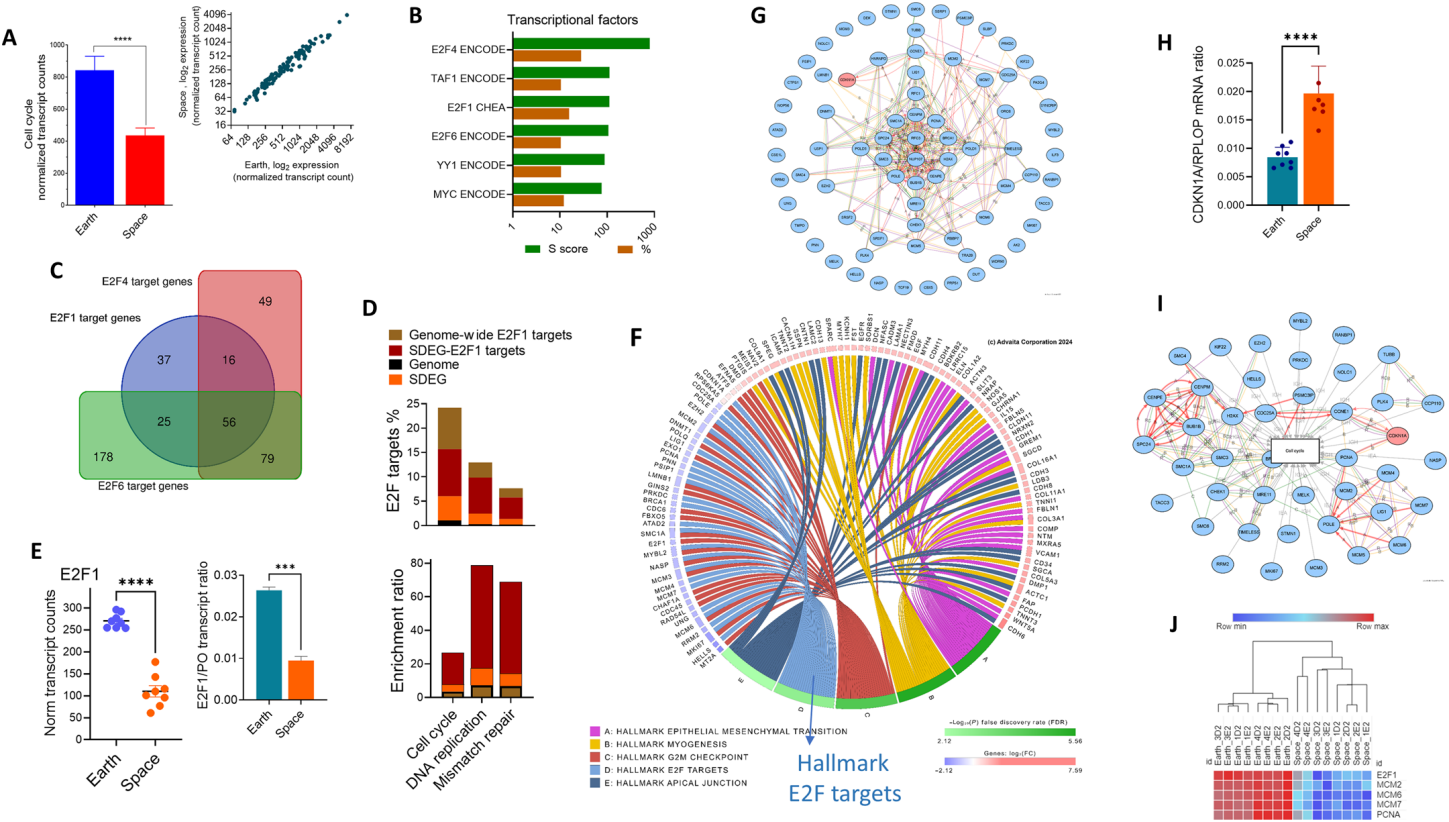
Cell cycle and E2F target identification and functional analysis of spaceflight-affected cellular gene expression. (**A**) A total of 118 cell cycle gene normalized transcript count data were obtained (left panel). Correlation plot of cell cycle gene expression between space and Earth. Means ± SEM (*n* = 8 per group). ****P < 0.0001 using unpaired *t* test with Welch’s correction (left panel) or Spearman’s test (right panel). (**B**) TF analysis using Enricher (Encode and CHEA databases). Data show ESS and % of the indicated TF target gene sets. (**C**) Venn diagram illustrating the common and distinct gene targets for the E2F family members (a total of 440 down-regulated SDEGs) representing the E2F members. (**D**) Proportion and fold enrichments for E2F1 targeted genes in relation to the indicated functional sets. The input gene sets for E2F TF assessment are 14,725, 858, 1029, and 134 genes representing THP-1 detected genome, E2F1 targets, down-regulated SDEGs, and down-regulated E2F1 target SDEGs, respectively. FDR < 0.0002. (**E**) The normalized transcript counts of E2F1 gene expression (*n* = 8 per group) are shown. ***P = 0.0002, Mann-Whitney test. Housekeeping *RPLP0*–adjusted ratios were obtained and presented similarly (right panel). (**F**) Chord diagram showing the top five significant hallmark pathways and their top core genes. Significant pathways and LFC for each core are shown as color legends. (**G**) Network analysis for protein interactions using the hallmark “E2F targets” was created using IPG. (**H**) Increased expression of the cell cycle inhibitor *CDKN1A* (p21). Normalized transcript counts were divided by their corresponding housekeeping transcript counts and presented as a ratio (means ± SEM, *n* = 8 per group). (**I**) Protein-protein interactions with the high confidence–linked cell cycle. (**J**) Gene expression heatmap for several E2F1 targets that are common between cell cycle and DNA replication groups. These genes represent normalized transcript counts (log_2_) with significant changes resulting from spaceflight (adjusted *P* < 10^−12^).

Cell cycle expressed genes (118 genes) as a group expression average were reduced because of the spaceflight exposure of the THP-1 monocytic cells by nearly half (1.9-fold, *P* < 0.0001; Fig. 9A, left panel). The gene expression abundance was highly correlative between Earth and space (Spearman correlation, *r* > 0.97, *P* < 0.0001; Fig. 9A, right panel). Analysis of high-ranking transcription factors (TFs), using the ChEA3/Decode TF network, identified members of the E2F family TFs, e.g., *E2F1*, as main players in the down-regulated gene group in spaceflight-exposed cells as opposed to Earth samples (Fig. 9B). There was a total of 440 SDEGs that are known to be targets of the E2F family: *E2F1*, *E2F4*, and *E2F6* (Fig. 9C). There were both overlapping and distinct genes in the E2F family (Fig. 9C).

The majority of the E2F family members are important regulators of the very same functions that are affected in the spaceflight cells, e.g., cell cycle and DNA replication. The E2F1 target genes constituted 36.2% of all the down-regulated SDEGs, and this was nearly the same proportion in the 783-gene MetaSpace Signature (37.6%). We focused on E2F1 because it is a major transcriptional activator of transcription during the cell cycle and other processes. We found that the cell cycle and DNA replication were highly affected in E2F1 targets in the spaceflight-exposed THP-1 cells compared to Earth control cells (adjusted *P* value <10^−9^; Fig. 9D). Compared to the human genome E2F2 target set (858 genes), there was 5.5-fold enrichment (FDR < 0.001) in the cell cycle pathway in the spaceflight-affected THP-1 cells. Similarly, the fold enrichment of DNA replication genes in the overall E2F1 target genome is 7.6, while the E2F1 target genes (DNA replication) expressed in the space-affected THP-1 cells were overrepresented eight times to an enrichment score of 61 (*P* < 1.85 × 10^−12^; Fig. 9D). Similarly, patterns of E2F and cell cycle pathways are also observed in datasets of I-4 multiome, I-4 cf-RNA, NASA Twin Study, and NASA GeneLab Meta-Tissue (data S5). For example, examination of I-4 data showed that E2F targets (hallmark module) are reduced in the CD16 monocytes (NES = −2.83, adjusted *P* < 1.31 × 10^−15^).

Among the cell cycle target E2F1 target genes is the E2F1 gene itself, which decreased by 2.7-fold (*P* < 0.0001) in the spaceflight environment (Fig. 9E). The top-scoring hallmark gene signature of the entire set of SDEG (up-regulated and down-regulated) confirmed enrichment in genes encoding cell cycle–related targets of E2F TFs (FDR = 0.002; Fig. 9F). There were 76 SDEGs comprising 38% of the hallmark pathway that were composed of 200 genes encoding cell cycle–related targets of E2F TFs. The mRNA abundances of all these genes were down-regulated, except for the critical tumor suppressor gene p21 [cyclin-dependent kinase inhibitor 1A (*CDKN1A*)], which was up-regulated (Fig. 9, G and H) and aligned well with spaceflight-affected underexpressed E2F-targeted genes (Fig. 9G). Network analysis demonstrates that the spaceflight-affected down-regulated group and the CDKN1A gene are of high-confidence gene-gene edge interactions and are significantly interconnected to the cell cycle process (Fig. 9I). The heatmap shows that the gene expression patterns in those genes that represent down-regulated E2F1 target genes in the spaceflight-exposed cells and also found commonly in the cell cycle and DNA replication functional clusters are *PCNA* (proliferating cell nuclear antigen) and the MCM (minichromosome maintenance) group members (Fig. 9J).

### The DNA damage signature of ionizing radiation is detected in the monocytic model

**Fig. 10.**
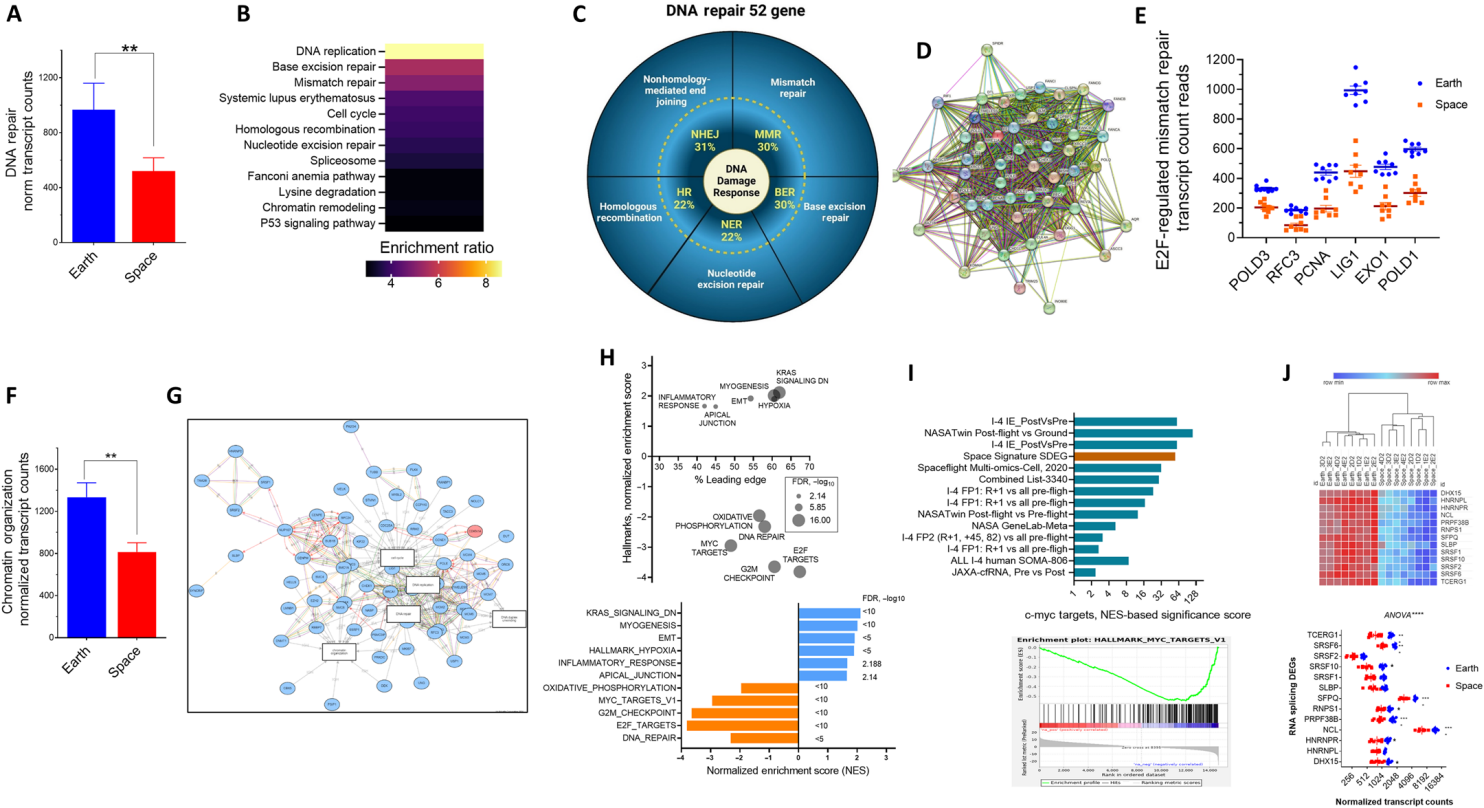
Mechanisms of spaceflight impact on DNA process–linked gene expression. (**A**) Expression of normalized transcript count data for down-regulated SDEGs linked to DNA repair (Reactome). The means ± SEM (*n* = 8 per group) are shown. ****P < 0.0001 using unpaired *t* test with Welch’s correction. (**B**) KEGG analysis of 1029 down-regulated SDEGs. The top enriched ratios (color legend) are shown. (**C**) Different processes of DNA damage repair are shown as % of the “DNA repair” pathway genes. (**D**) Protein-protein String analysis of 50 mapped gene products encoded by DNA repair SDEGs. (**E**) Six mismatch repair genes that are E2F1 targets and down-regulated in spaceflight versus Earth samples. (**F**) Normalized transcript count expression plot of the chromatin organization pathway in down-regulated SDEGs. Data are the means ± SEM (*n* = 8 per group). **P = 0.002 using Student’s *t* test with Welch’s correction. (**G**) Network analysis of four GO biological processes mapped to SDEGs that affect the DNA. The network with a high (>70%) confidence was built using the IPG algorithm; the nodes represent the E2F target genes (67 hallmark gene set), and the edges represent regulatory interactions between genes. (**H**) GSEA of our SDEGs was performed with ranked *t* test values. Hallmark enrichments are shown with NES (upper and lower panels) and % leading edge genes (upper panel). The symbol size corresponds to −log_10_ FDR. (**I**) The c-myc target hallmark is common in many spaceflight mission data. Several mission database subsets were queried using the SOMA browser or obtained from the indicated supplementary data. Data shown are the absolute value (NES) × −log_10_ adjusted *P* value. Lower panel: GSEA output (example) for the c-myc target hallmark obtained using our SDEGs. (**J**) Heatmap of 12 underexpressed SDEGs obtained by intersection of c-myc–regulated genes (45 as detected by TF Enricher analysis) and also enriched in the RNA splicing pathway (KEGG/Reactome, FDR < 0.001).

Exposure of cells and tissues to ionizing radiation is known to affect both the cell cycle and DNA repair processes. Our monocytic cellular model during spaceflight indicates that the DNA repair gene group is altered (Fig. 2, G and H). There was a significant reduction in many DNA repair genes (*n* = 52 genes, nearly twofold reduction) (*P* < 0.0001, Fig. 10A). We further performed KEGG analysis to identify the specific mechanisms involved in these down-regulated genes. KEGG pathways showed that different mechanisms of DNA repair are compromised (Fig. 10B). These pathways have both distinct and overlapping members representing the following enriched pathways: base excision repair, mismatch repair, homologous recombination, nonhomologous end joining, nucleotide excision repair, and Fanconi anemia; they were down-regulated in the spaceflight-exposed THP-1 cells (Fig. 10, B and C). The DNA repair genes are highly interactive (*P* value = 0) at the level of protein-protein interactions, which was 893, while the expected number of interactions was 156 (Fig. 10D). As a control, 52 THP-1 random genes show no significant interactions (*P* = 0.87). The DNA repair genes were enriched in the E2F target family (67%), with the mismatch repair pathway as the prominent group (FDR = 7.29 × 10^−04^) in the E2F1 targets of the down-regulated SDEGs when compared to Earth controls (Fig. 9D). There were six mismatch repair genes that are both E2F1 targets and down-regulated in spaceflight samples with a mean of 0.47-fold ± 0.03 SEM (*P* < 0.0001) compared to Earth samples (Fig. 10E). In general, most of the down-regulated genes due to the spaceflight cellular effects are associated with pressure on the DNA processes: DNA replication, DNA repair, chromatin organization, and DNA unwinding (Fig. 10, F and G). There were 341 interactions mapped in this network (Fig. 10G).

### c-myc–regulated mRNA splicing is selectively down-regulated and is a common spaceflight signature

The c-myc TF targets are found enriched by threefold (FDR < 1.66 × 10^−13^) in the space down-regulated gene group (Fig. 9B). According to CHEA and EnCODE databases, there were 76 and 183 myc-targeted genes, respectively, that were underexpressed in the space samples, corresponding to nearly 12% of all genome Myc targets. We obtained NESs from GSEA using the Molecular Signatures Database (MSigDB) hallmark module to compare our SDEGs with other missions’ data (Fig. 10H and data S3). We found that c-myc targets are commonly enriched as a down-regulated group in the DEGs of multiple spaceflight multiomics datasets, including our set here (Fig. 10I). Moreover, this “common spaceflight feature” is found in multiple cell or tissue types, including blood cells, skin, Meta GenLab multitissue mouse data, cultured cells, and cf-RNA.

The most represented pathway of the myc targets (45 genes, common sets of CHEA/EnCODE) in the down-regulated SDEGs was mRNA splicing, representing 12 different genes; among those are four serine/arginine–rich splicing factor family members (Fig. 10J). The expression of these genes was reduced by 40 to 47% (adjusted *P* < 10^−12^) in the spaceflight samples. Unlike E2F1, whose mRNA expression itself is down-regulated in the spaceflight-exposed cells, c-myc expression is not, indicating mechanisms such as posttranslational modifications that may reduce c-myc activity. As with other datasets, mRNA splicing is one of the top affected pathways in spaceflight with a negative NES (i.e., the majority is down-regulated). For example, an NES of −3.72 (−log_10_ of *q*-value = 40) in the Reactome_mRNA pathway in astronauts’ blood CD16 monocytes in fp2 I-4 data and an NES of −2.89 in the NASA Twins Study (*q* = −23.8) were reported (SOMA browser).

The use of GSEA with the hallmark database uncovers two important gene sets, one with a negative NES score, oxidative phosphorylation, which has been shown in the other multiome datasets (Fig. 10H). Expectedly, the well-documented inflammatory response with a positive direction was seen in GSEA hallmark analysis (Fig. 10H).

### Astronaut illness and organ toxicity mapping by ML in spaceflight-affected cells

Certain conditions, particularly the cardiovascular and myopathic disease annotations, were identified with other analyses (Figs. 3F, 4I, and 7B). Using ML disease algorithms, several other disease pathways were observed with potential similarities to some of the symptoms encountered by astronauts. When the THP-1 random gene list was used, the identified diseases were not annotated, at least statistically (fig. S5A). Other diseases identified by IPA were similar to those identified by iPathwayGuides (IPG) analysis, but a notable disease category was identified, spermatogenic failure (fig. S5B), with 38 SDEGs of the 68 disease pathway genes (fig. S4C). Using the toxicological disease database, the ML IPA program identified two additional important health conditions, kidney/renal and liver damage (fibrosis and cirrhosis; fig. S5D), in addition to the cardiac toxicity gene group that was generated from cardiac abnormality analysis (Fig. 3). These three disease states are associated with drug-induced toxicity. Using the IPG program, a disease subcategory was identified as renal tubular acidosis, which can cause renal/kidney damage (fig. S5E).

## DISCUSSION

Spaceflight missions allow the biomedical research community to study a chronic disease as an accelerated process to explore the associated molecular changes and find therapy drugs. Recently, several multiomics studies have demonstrated appreciable changes at the cellular and molecular levels that occur during spaceflight (*42*). Our data are in agreement with several spaceflight-affected broad-level pathways, such as the sensory system, muscle system, oxidative phosphorylation, and DNA damage, but are found here coupled with specific molecular-level alterations. The magnitudes of altered pathways and specific DEGs are different among the space omics studies, including ours. Moreover, our model reveals molecular alterations during exposure to spaceflight conditions, particularly those related to the cardiovascular, neuronal, and sensory systems. Specifically, we identified mechanistic networks associated with vision abnormalities, sleep disturbances, and movement disorders, including retinoid metabolism and transport, CREB signaling, and GRS. These pathways were, at least statistically, mapped to various diseases or health issues similar to those that can occur during spaceflights.

THP-1 is a highly plastic myeloid cell line capable of modeling immune reprogramming and stress-response transcriptional activity. The plasticity of the monocyte-macrophage lineage enables it to adapt to different tissues and environments and to respond to insults. As an example, with THP-1 differentiation into macrophages by treatment with a single agent, phorbol 12-myristate 13-acetate, more than 5000 DEGs (more than twofold, *P* < 0.05) were documented (*43*). Also, a large number of expressed genes, 3348, were reported to be differentially expressed between at least two tissue-resident macrophages or monocytes (*22*). As with tissue homing and adaptability, spaceflight conditions induce chromatin changes and subsequent gene expression reprogramming (*1*, *44*). This feature can help explain, at least partially, how the THP-1 monocytic cells respond highly to the space microenvironment and may offer an advantage for spaceflight studies.

Compared to previous omics studies, which used different cellular and physiological models (*35*, *37*), our model comprises only one cell line that reflects multiple alterations in signaling and disease pathways. These findings may suggest that fundamental spaceflight-responsive mechanisms may be conserved across different cell types and tissues. However, this does not mean that THP-1 can model the physiology of specific organs and other tissues. An advantage is that the THP-1 cell line has a stable, homogeneous genetic background (*19*) compared to individual variations in astronauts’ body cells, fluids, and other models. An important feature in the study experimental design, when compared to other omics studies with astronauts’ blood, live mice, and live cell line deployment, is that the launch and retrieval of frozen cells and RNA*later* samples were respectively maintained at −80°C. This approach should remove many of the spaceflight confounding environmental effects such as vibration, hypergravity during launch, nutrient uptake, and temperature variations. Thus, the molecular changes and pathways observed in our omics may be stronger toward microgravity and radiation as opposed to other environmental factors. However, future work is needed to find whether microgravity is the major factor, for example, by using simulated microgravity or gravity control at the ISS.

We noted that in the analysis of our model and other multiomics data (including monocyte or peripheral blood mononuclear cell data), the neurotype cell–associated gene expression profile and the brain macrophage-monocyte type (microglial) were positively enriched (Fig. 8, B and G). There was overexpression of Spalt-like TF 1 (*SALL1*) in our SDEGs (21-fold, −log adjusted *P* < 20), which is selectively induced in microglial cells (*45*). Several neuronal system diseases were related to GRS, a molecular pathway that is highly enriched in our spaceflight SDEG set (135 genes). This pathway involves a response to glutamate, a key neuromodulator that controls neuronal communication and synaptic functions; it involves an array of glutamate receptors primarily on the membranes of neuronal and glial cells (*46*). The glutamatergic signaling pathway as shown by ML/artificial intelligence in our study is associated with movement and coordination disorders (Fig. 7). These observations, at least hypothetically, are relevant to astronauts and life in space, given that sensory-motor and cognitive control dysfunctions are thought to occur during long-duration spaceflight (*47*, *48*). Spaceflight conditions can affect bodily changes related to the symptoms of space motion sickness, spatial disorientation, and movement difficulties (*47*, *49*). Spatially resolved multiomics with mouse brains in response to spaceflight demonstrated effects on neurogenesis, synaptogenesis, synaptic transmission, and astrocyte function (*50*).

Cardiovascular abnormalities are reported in spaceflight astronauts, in which extended exposure to microgravity can lead to fluid shifts toward the head, plasma volume reduction, modified arterial pressure, cardiac muscle atrophy, and arrhythmias (*51*, *52*). Radiation exposure contributes to coronary artery degeneration and aortic and carotid stiffness, which, with long-duration spaceflights, can accelerate atherosclerotic disease (*52*). Our data show that statistical enrichment and ML models revealed associations with several adverse cardiovascular conditions, largely grouped into cardiac conduction and striated muscle contraction gene groups and particularly associated with cardiac arrhythmias and cardiomyopathy, respectively. Cardiac arrhythmias can be particularly observed with longer-duration spaceflights, as noted by NASA reports, and involve prolonged QT changes (*53*). The most common form of cardiac arrhythmias is atrial fibrillation, which was among the top identified diseases in our SDEGs, and its prevalence in astronauts is suggested, although this requires further evidence. The cardiac *RYR2* was the most abundantly induced among SDEGs in our spaceflight-affected THP-1 cells (Fig. 3F). This gene is involved in left ventricular abnormalities, which are significantly annotated to our SDEGs. It increases cytosolic free calcium and subsequent cardiac changes (*54*). In mice, simulated microgravity–induced cardiac remodeling was suggested to be associated with RYR2-increased phosphorylation (*55*).

Muscle atrophy is one of the most observed abnormalities for decades encountered in spaceflight, mostly caused by microgravity. In our SDEG set and several RNAomic datasets, muscle system alterations are observed in astronauts’ blood cells, including CD14 and CD16 monocytes (e.g., I-4 study and NASA Twins Study), and other organs/tissues. Specifically, the (striated) muscle cell differentiation pathway was identified as the top up-regulated GO (gene ontology) biological pathway in the spaceflight signatures of the I-4 astronaut’s blood cells (*35*). The striated muscle cell differentiation pathway was observed, particularly with dilated (and hypertrophic) cardiomyopathy, indicating the fitted relationship between spaceflight-affected gene expression in cardiac muscle conduction pathways and the probably expected cardiac abnormalities in long-duration flights. Cardiomyopathy-associated alteration in *TPM2* gene expression in our spaceflight-exposed THP-1 cells (Fig. 4) mirrors the relationships between *TPM2* genetic variant–affected muscle development and function changes that lead to a number of congenital myopathies, including nemaline myopathy, cap disease, distal arthrogryposis, and hypertonia (*56*, *57*). TPM2 is β-tropomyosin, an actin-binding protein, that is central to muscle and cardiac contraction (*58*), and thus, alterations in its abundance or activity can have marked effects.

Sleep disturbances due to circadian rhythm changes are well noted in astronauts and reflected in the pathway enrichment in recent spaceflight multiomics studies, even with different tissues and cell types, not only the eyes (*37*, *59*). Notably, these observations confirm our THP-1 SDEGs. One of the up-regulated circadian rhythm genes in our SDEGs is *NPAS2* (neuronal PAS domain protein 2), one of the two genes that are commonly expressed in all mouse tissues (*59*). Circadian rhythm-sleep disorders can be managed by melatonin, which is found safe and beneficial in space flights (*60*, *61*). Moreover, it is proposed that it could be used as a drug for the prevention of bone loss during spaceflights, as it is intriguing to know that spaceflight can cause the activation of osteoclasts and contribute to bone loss, while melatonin inhibits this aberration (*61*, *62*). Sensory systems (visual, hearing, taste, vestibular, and olfactory) are known to be disturbed in astronauts during spaceflight, and differential gene expression in our SDEG signatures related to the underlying processes is manifested in our cellular model.

Most of the enriched pathways in which the majority is down-regulated SDEGs concur with the recently reported in vitro and in vivo data (*35*), which include cell cycle and mismatch DNA repair. There were two master TFs, E2F and c-myc, that were strongly associated with cell cycle and RNA splicing. Careful examination of NASA GenLab mouse multitissue, I-4, and other data demonstrates the same trend and patterns of these enriched pathways. The E2Fs are involved in the regulation of the cell cycle, DNA replication, and proliferation (*63*). The down-regulation of the E2F-mediated pathway and cell division has been previously reported under simulated spaceflight conditions and may result from the combined effects of radiation and microgravity (*64*, *65*). We identify a common feature among many of the spaceflight datasets, including our SDEGs, which is c-myc targets (Fig. 10), and they are enriched in both the cell cycle and RNA splicing. DNA damage, as well as the decreased expression of DNA repair genes involved in mismatch repair, base excision repair, and nucleotide excision repair was observed in both spaceflight and simulated microgravity (*10*, *66*). DNA damage is a feature of ionizing radiation that can be encountered in space. Space radiation is considered a major risk to astronauts; exposure increases the risks of cancer, damage to the central nervous system, and tissue degeneration (*15*). Oxidative phosphorylation is another pathway that has been shown to be altered (reduced) under both simulated and spaceflight conditions (*35*, *67*). We have also seen this pathway as one of top significant hallmarks identified by GSEA (Fig. 10H).

As space-based research faces limitations because of its technical and costly logistical challenges, it was necessary to cross-validate our findings with existing space omics data. In general, space omics studies are based on computational enrichment analysis; these associations do not imply direct functionality, especially when comparing pathways across tissues. The spaceflight-affected disease enrichment analysis, including ML tools, identified annotated diseases and conditions (Figs. 3F, 6D, 7B, and fig. S5) similar to those encountered with spaceflight travel. However, the monocytic-macrophage model does not replicate complex tissue interactions. Nevertheless, our findings on disease associations may provide hypothesis-generating opportunities to explore with other tissue-specific models. Potential next steps could be further researched on specific genes or altered pathways that have not been explored in this study.

Overall, our study provides a comprehensive framework for spaceflight-affected molecular alterations, including enrichment pathways and specific molecular signaling networks. These are associated, at least computationally, with diseases and conditions that exhibit symptoms similar to those experienced by astronauts, depending on the duration of the mission. These findings were uncovered by a cellular model, which uses THP-1 as a highly plastic myeloid cell line with features of genetic stability, immune reprogramming, and stress response capability. These features are advantageous for future research in space environments or simulated conditions. The model can be used as a disease-accelerated platform, as many molecular alterations, including specific signaling pathways associated with disease, can be targeted by specific drugs.

## MATERIALS AND METHODS

### Cell line

Microgravity can be challenging for cell adhesion in space, which might alter cell behavior, and given the variability of operational schedules and crew time constraints during the AX-2 mission to the ISS, the nonadherent suspension THP-1 cell line (TIB-202) was chosen as a model cell line for its suitability and adaptability. The fresh cell batch was obtained from American Type Culture Collection (Rockville, MD) and cultivated in RPMI 1640 medium (Sigma-Aldrich, R8758), supplemented with 2 mM l-glutamine, streptomycin (0.1 mg/ml), penicillin (100 IU/ml; Sigma-Aldrich, G6784), and 10% heat-inactivated fetal bovine serum (Sigma-Aldrich, F0926). The cells (million per milliliter) were maintained at 37°C in a 5% CO_2_ incubator in 10 ml of total media. To test cell viability, a 10-μl aliquot was diluted with 10 μl of trypan blue solution, and the vital cell number was counted (viability >95%). The experiments were carried out using passage number five.

### Experimental verification test (EVT)

During the final design review, validation tests were carried out at BioServe Space Technologies in Colorado, US, which assisted the scientific team in facilitating a ground test of the AX-2 mission cell experiments. The experimental verification test (EVT) was thoroughly detailed, outlining the equipment and materials to be used on board the ISS to ensure that they met all NASA requirements, such as safety, equipment’s availability, and access. Different simulations were performed, including cell and reagent storage, transportation temperatures during the ascending and descending of spaceflight, cell viability throughout the experiment duration, and the time requirement for each event requiring crew time. Subsequently, a proposed design and configuration were performed as a complete run (Earth to space to Earth) and found robust, as detailed in the following sections. Experimental procedures and operations were transformed into a communication protocol for the crew. The first Arab (Saudi) female astronaut, who herself was a well-trained laboratory specialist at King Faisal Specialist Hospital and Research Centre, Riyadh, in cellular and molecular biology, carried out most of our experiments at the ISS.

### Launch preparation

After the EVT, cells were prepared to be transported to Kennedy Space Center (KSC), Florida. Cryovials, i.e., freezing cell vials (Sigma-Aldrich, V5005) prewrapped in Velcro for stationary stability aboard the ISS, were prepared. Each contained 10 million cells in 0.5 ml of freezing media, containing 10% dimethyl sulfoxide (Sigma-Aldrich, S-002-D) and 20% heat-inactivated fetal bovine serum; each vial was allocated to seed four wells of two 48-well plates, and one extra vial was used as a backup. Cryovials were stored in liquid nitrogen before moving them into a dry ice container to be transported. Once in KSC, they were moved back to liquid nitrogen before spaceflight.

Three days before launch, the payload launch kit was prepared at KSC to be transported on board the SpaceX Dragon capsule on the Falcon 9 rocket. Cells in cryovials were transferred from the liquid nitrogen tank, packed into labeled Bitran bags, and carried in a dry ice container. On board the dragon capsule, they were stored and launched at a −80°C stowage vessel. The complete medium was packed as four 10-ml syringes filled with 7 ml of medium; the syringe was filled using a closed-system plunger tool to ensure sterility. These were sealed in a Bitran bag along with 1000- and 200-μl pipettes and tips. The eight RNA*later* reagent (Sigma-Aldrich, R0901) tubes for RNA preservation were prefilled and packed in a Bitran bag for sample collection and return. The supplies destined for the ISS were fastened to guarantee their safety and functionality under the microgravity conditions of space. Specialized packaging was used to reduce the risk of contamination during transit, including protection against factors like vibration, temperature fluctuations, radiation, and hypergravity encountered during launch to ensure that the supplies arrived intact and ready for use upon arrival to the ISS—although these factors are minimized by the fact that the supplies are shipped frozen at −80°C.

### Cell culture on board the ISS

Cells from the cryovials were retrieved from a −80°C stowage vessel into Life Science Glovebox (LSG), where they were thawed in a prewarmed Flexboy pouch at 37°C for 2 min. Each vial was allocated to seed four wells of two 48-well plates with eight wells, four wells per plate. The experiments were handled on two different days, subsequently called batch I and batch II. Before pelleting the cells, 1 ml of complete media was gently layered onto the cell suspension, followed by centrifugation at 2000 rpm for 5 min using an Eppendorf Mini-Spin 5452 microcentrifuge. The supernatant was gently removed, and the cell pellet was suspended in 1 ml of complete media. This step was repeated twice to remove any remaining dimethyl sulfoxide. Volumes of 250-μl of cell suspension were dispensed into each well of Corning polystyrene 48-well plates at a density of 2.5 million per well. The plates were then sealed inside two vented bags to prevent potential fluid spillage and allow cells to breathe. The plate was then placed in a humidified incubator at 37°C with 5% CO_2_ and left to acclimatize for 48 hours. The plates were removed from the incubator and the bags, and 250 μl of cell suspension from each well was transferred to 1-ml RNA*later*-prefilled tubes. The RNA*later* samples were mixed and left at room temperature for 24 hours, following the manufacturer’s instructions. These samples were stored and transported back to Earth at −80°C aboard the Dragon spacecraft. In essence, the cells and extracts were all maintained at −80°C throughout spaceflight.

### Postflight ground control sample processing

To minimize discrepancies between ground control (Earth) and spaceflight (space) experiments, the ground control experiments were conducted after the completion of spaceflight when all the operation rosters were obtained. The experimental procedures and operational protocols at the ISS and launch/descend conditions were documented in detail. The cell culture preparation, sample collection, and storage procedures were exactly duplicated using the same hardware, exact protocols, and procedures to maintain uniformity in experimental design, allowing reliable conclusions about the influence of spaceflight on gene expression.

### RNA samples and cDNA synthesis

There were 16 RNA samples representing two batches (at two different and consecutive days) constituting eight spaceflight and eight ground control samples. These samples were part of a larger project of nearly 200 samples representing multiple projects at the ISS. Initially, samples preserved in RNA*later* were centrifuged for 5 min at 2000 rpm to pellet the cells, and the supernatant was fully aspirated. Total RNA was purified using the automated QIAsymphony RNA system (Qiagen, Germantown, MD). Approximately 500 μl of RLT lysis buffer was added to each cell pellet, and RNA was processed according to the manufacturer’s recommendations to yield DNA-free RNA. We opt to use the ultralow-input RNA-seq approach, which comprises SMARTer Stranded Total RNA-Seq Kit v3 Pico Input (Clontech/Takara Bio Inc., CA). This method, suitable for a low yield of RNA, particularly those of spaceflight samples, produced high-quality transcriptomic data and depth (as in our case, details in the next library and sequencing quality control. The ultralow methods have been developed to amplify full-length transcripts with minimal bias selectively, and the quality and sensitivity of these results are similar to those of standard RNA-seq experiments (*68*). The average DV200, a more reliable determinant of RNA quality upstream of library preparation (*69*, *70*), is >50% for all samples. This optimized protocol offers the best performance for RNA samples with DV200 > 50%, and even good-quality libraries have been obtained from RNA with DV200 values as low as 25% (Clontech Manufacturer notes). The switching mechanism at 5′ ends of the RNA template was performed to generate the full-length cDNAs. Before cDNA conversion, the RNA was fragmented to a size suitable for sequencing on Illumina platforms. The cDNA was synthesized with an integrated step of the removal of cDNAs derived from ribosomal RNA.

### Library preparation and library quality control

Adaptors were added using transposase, followed by low 12 cycles of amplification [polymerase chain reaction (PCR)] to enrich and add index to the cDNA fragments. The amplified tagged cDNA was purified and subjected to the final RNA-seq library amplification using the Illumina Nextera XT library. The fragments were enriched via a second round of amplification using primers universal to all libraries. The PCR products were purified again to yield the final cDNA library, which now contains sequences that allow clustering on Illumina. Sequencing libraries were validated using the Agilent TapeStation and quantified using the Qubit Fluorometer and quantitative PCR (KAPA Biosystems, Wilmington, MA). The library size distribution was evaluated and found to be of high quality; the electropherogram pattern demonstrates a broad single peak with no artifacts (example in fig. S5). The expected average sizes of 373 to 420 base pairs at region 100 to 1000 boundaries were obtained. The sequencing libraries were multiplexed and clustered on a NovaSeq 6000 flow cell, targeting ~20 million reads per sample. After clustering, the flow cell was loaded on the Illumina instrument. The samples were sequenced using a 2-by-150 paired-end configuration.

### Sequence processing and quality control

Quality control metrics were assessed at each step to ensure the accuracy and reliability of the results obtained from the sequencing data. Raw sequence data generated from the sequencing were converted into FASTQ files and demultiplexed using Illumina’s bcl2fastq 2.17 software. One mismatch was allowed for index sequence identification. After raw data quality assessment, sequence reads were trimmed to remove possible adapter sequences and nucleotides with poor quality using Trimmomatic version 0.36. The trimmed reads were mapped to the reference genome available on ENSEMBL—*Homo sapiens* GRCh38—using STAR aligner version 2.5.2b. BAM files were generated as a result of this step. The sample sequencing statistics for each sample included the size (number of reads and yields in megabase), mean quality score, and % bases >30 [all were within the acceptable criteria (*71*); see data S6]. The average of the reads is excellent and was ~47 million, the mean quality score is >35, and the read quality score is more than 90%. Only unique reads within exon regions were counted, mapped to the reference genome, and aligned by bioinformatics to generate hit count exons/genes. Moreover, the statistics of mapping the reads to the reference genome for each sample, including total reads, mapped reads, % mapping, unique mapped reads, and % unique mapping, are excellent (data S7).

### RNA-seq analysis

Unique gene hit (read) counts were calculated by using featureCounts from the Subread package version 1.5.2. The read counts were summarized and reported using the gene_id feature in the annotation files. The read count table was used for downstream differential expression analysis using the DESeq2-based approach. The read counts (a total of 57,000 genes) were obtained and filtered from genes with low counts, where the mean row count is less than a defined threshold of 10 counts, resulting in nearly 21,000 genes. These filtered read counts were normalized by median ratio normalization, a robust method that yields fewer FDRs (*72*) where the weighted means of gene expressions are considered for both conditions (space and Earth). Batch correction was also performed using the Combat algorithm (Fig. 2A), allowing the inclusion of all 16 samples of the two batches for analysis. The Differential Expression Browser (https://nasqar2.abudhabi.nyu.edu/DEBrowser/) was used to find DEGs using DESeq2 (*73*) with the following parameters: Wald significance tests and the nonparametric fit of dispersions to the mean intensity. The adaptive shrinkage method using empirical Bayes for LFC shrinkage was used to improve the estimation quality in differential expression analysis and generate reliable LFC estimates (data S1). The DESeq2 output is now normalized counts with adjusted *P* values, LFC, and Wald’s test statistic values. Gene types were obtained for the resulting normalized read count annotation using Ensembl BioMart. PCA plots of the 16 samples after normalization (Fig. 2A) showed that PCA1, with a notable difference of >48%, separated the space and Earth groups, whereas PCA2 (minimal <10%) separated within the group samples (fig. S1).

### Derivation of DEGs using SS and *k*-means clustering

SSs were calculated according to the following function

$$\text{SS}={\text{Log}}_{2}\;\left(\text{fold change}\right)\times -{\text{Log}}_{10}\;\left(\text{adjusted}\;P\;\text{value}\right)$$

This function has been previously validated (*31*). Genes were ranked according to their SS, where a positive score is counted as up-regulated, and a negative score is down-regulated gene expression. The generated data were used as an input list for *k*-means clustering to produce two-mean clusters for each positive score group and negative score group. *k*-Means clustering was performed using the JMP program (SAS); *k*-means clustering is a form of the expectation (cluster means) maximization algorithm that assigns points to the closest cluster. Subsequently, the significance thresholds were obtained at the intersection of the two clusters of each group of the up-regulated and down-regulated groups. Our inclusion criteria for compiling significant (spaceflight) differentially expressed genes (SDEGs) were to include genes with an SS greater than the obtained thresholds.

### SDEG visualization and heatmap

Normalized LFCs from DESeq2 output were processed/visualized as a heatmap by Morpheus (https://software.broadinstitute.org/morpheus). The normalized LFCs were processed by two-way clustering (single linkage, with the Euclidean distance algorithm), and the color scale depicts the degree of change where red indicates maximum expression and blue represents minimum expression. A symmetric Venn diagram was generated to calculate group intersections using an online web–based tool (https://bioinformatics.psb.ugent.be/webtools/Venn/). Normalized transcript counts for SDEG data were visualized using different graphs, as indicated in the legend, using Prism GraphPad version 9.5.1. Chord visualization was produced from IPG software, in which top to lower connections indicate gene memberships for each pathway, including overlapping and common genes. Other visualizations were indicated in figure legends.

### Background and random list controls for enrichment analysis

We applied stringent criteria for pathway and functional enrichment and network analysis by using a background reference list derived from all detected protein-coding genes in THP-1. This is important because, in many programs, all annotated protein-coding genes (genome) are often used as default. Thus, the use of a THP-1 background is crucial to ensure the accuracy and biological relevance of the results compared to the usage of a protein-coding genome. When creating random lists, we used the same size (number of genes) as with the input list (e.g., SDEG), the same type (protein-coding gene as defined in Ensembl), and the same background (THP-1 cells). In certain analyses, particularly cell types, the random list was generated similarly but with the protein-coding genome background. This approach was confirmed robust, as analyzed in Results. Random lists were generated by using the RandomSampler program at biotool.fr. Data S8 shows the description of the background and random lists used.

### Broad enrichment analysis: Reactome and hallmark pathways

WEB-based GEneSeT AnaLysis Toolkit (www.webgestalt.org/) was used to calculate the enrichment score and the adjusted *P* values. The organism of interest was *H. sapiens*, the background THP-1 list was manually uploaded, and ORA analysis was used. Fisher’s exact test (significance level, FDR < 0.05) and Benjamini and Hochberg multiple testing to adjust *P* values were performed. To find broader categories for both the Reactome and hallmark pathways, we used the redundancy reduction algorithm “weighted set cover,” which allows simultaneous consideration of both gene set size and significance. This algorithm results in the fewest number of gene sets that cover all DEGs in the enriched sets, with the most significant sets generated (*74*). The enrichment SS (ESS) that combines both the odds (enrichment) ratio and the adjusted *P* values was used as follows: ESS = (fold enrichment × −log_10_ adjusted *P* values or FDR).

### Molecular pathway analysis

Data were analyzed to identify significantly affected molecular pathways, e.g., KEGG pathways. This was done using programs IPG by Advaita Bio Corporation and IPA (version, Qiagen Inc.) software canonical pathways (Qiagen). Advaita’s proprietary “Impact Analysis” method comprises two types of evidence: (i) the ORA of DEGs in a given pathway and (ii) the perturbation of that pathway computed by propagating the measured expression changes across the pathway topology and then combined into an overall pathway score by calculating a *P* value using Fisher’s method with FDR or Bonferroni correction (*75*). The underlying pathway topologies, composed of genes and their directional interactions, are obtained from the KEGG database. For the IPA program, Fisher’s exact *t* test was also implemented on the IPA canonical pathways to calculate overlap ratios and adjusted *P* values.

### Disease-pathway relationships using ML programs

We used both annotation-based and ML algorithms for disease associations, MOET disease ontology and ML IPA, respectively. The MOET disease ontology module used the Human Disease Ontology database. The statistical test used in this module is a hypergeometric test with Bonferroni-corrected *P* values and odds ratios for the overrepresented terms. The ML disease algorithm, a part of the IPA program, was also used. The algorithm tries to causally connect gene sets to the disease and reports the ratios and *P* values on the basis of Fisher’s exact test. IPG software was also used, which uses the ORA approach to compute the statistical significance of observing at least the given number of DEGs. The *P* value is computed using the hypergeometric distribution and corrected for multiple comparisons using FDR and Bonferroni correction.

### Cell-type and deconvolution analysis

Cell mapping profile comparisons between our THP-1 control samples (*N* = 8) and normal blood monocytes (*N* = 8) were performed. The latter dataset contains raw counts of eight normal monocyte expression data that were downloaded from Expression Atlas (GSE77598) using healthy donor controls of the experiments. Comparisons were made using Dseq2 with Limma cross-normalization. The Meta-predict computational deconvolution method (BigOmics Analytics, Switzerland) was used against two reference databases of immune cell expression: DICE and the single-cell sequencing LM22 datasets. The cell profiles were visualized as dot blots using BigOmics Analytics. Meta-analysis for SDEG versus random THP-1 list was performed using IPG by using MSigDB with C8 module collection, which contains gene sets with curated cluster markers for cell types identified in single-cell sequencing studies of human tissues. Similar to the above analysis, the *P* value is computed using hypergeometric distribution and corrected for multiple comparisons using FDR or Bonferroni correction. For cell-type analysis, the appropriate background is the protein-coding genome. For visualization, the UMAP and the multivariate bubble plot (JMP software) were used.

### External datasets for further validation of the SDEG signatures

We used different external datasets for further validation/confirmation of our data analysis. Much of the information was retrieved from the SOMA, particularly multiomics analysis of astronauts, including the crew blood samples from the I-4 and Axiom-1 missions. It also included JAXA cf-RNA and GenLab metasets of mouse tissues. These data were accessed via https://soma.weill.cornell.edu/apps/SOMA_Browser/ and supplementary data from the original studies. Space-X I-4 spaceflight was the first mission (3 days) that carried four civilians to orbit Earth; we used postflight and preflight (fp2 and fp4) data (*35*). The I-4 cf-RNA data were also queried from the SOMA browser. The NASA Twins Study is from the blood cells of one astronaut who was monitored before, during, and after a 1-year mission on board the ISS, while his twin sibling was monitored on Earth simultaneously (*76*). The JAXA plasma cf-RNA study comprises RNA-seq from RNA extracted from the plasma collected from six astronauts prior, during, and post spaceflight on the ISS (120-day long duration). Data S9 contains all the pertinent datasets. Comparisons of the above data with our SDEGs were performed on the basis of GSEA and the overall protein-coding genome background to allow for a comparable analysis.

### Derivation of combined and common spaceflight signatures

We derived two different metasets comprising spaceflight omics data for further validation/confirmation of our SDEG analysis. First, a combined list of *n* = 3340, called MetaMission, comprises nonredundant DEGs (protein-coding) obtained from the combined list (*N* = 5886 DEGs) of the following datasets: (i) The NASA GeneLab Meta-Tissue set represents human homologs of DEGs (*n* = 1962) from the mouse RNA-seq data of 27 datasets encompassing 10 different mouse tissues (adrenal glands, colon, eye, kidney, liver, lung, skin, muscle, spleen, and thymus) and obtained from the I-4 study. (ii) A total of 1875 JAXA cf-RNA study DEGs are processed from the RNA-seq data using the plasma of six astronauts’ blood and obtained from GeneLab (GLDS-530/OSD-530). (iii) The multicell RNA set is combined from the DEGs of the following datasets: GLD-13, activated T cells, GLD-52, human endothelial cells (human umbilical cord endothelial cells), GLD-114 human fibroblasts, and GLD-174 follicular hair (*37*). (iv) A total of 806 DEGs are obtained from the I-4 omics study using RNA-seq data of CD14+ and CD16+ monocytes (*35*).

The second list comprises the common genes between the combined MetaMission and our SDEGs, resulting in 873 genes collectively referred to as the MetaSpace Signature. Data S10 contains the sources and gene datasets of the external omics data resources. The background used was all protein-coding genomes, given that the combined and common gene groups were derived from multiple tissues.

### GSEA

We ran GSEA on all the 14,724 detected expressed genes that included the SDEGs for the comparison with various NASA GenLab, I-4, and other mission datasets using wgstat web–based analysis with a ranked gene list. The ranked gene list was ordered by Wald’s test statistic values obtained from DESeq2 analysis, whereas the Wald statistic LFC was divided by its standard error. The pathway’s NES, gene size (number of leading-edge IDs), and corrected *P* values were obtained from GSEA using Reactome, KEGG, and MSigDB hallmark databases. Redundancy was removed using the weighted set cover algorithm. Positive and negative NES values depict the direction/magnitude of the enrichment. FDR values are reported with at least less than 1% (<0.01).

### Other statistical methods

Depending on the data, standard statistical tests were used. For example, Student’s *t* test with Welch’s correction or Mann-Whitney tests was used to identify the difference between two columns. Spearman correlation was used to calculate between two different columns. Two-way analysis of variance (ANOVA) with Šídák’s multiple comparisons test was used when comparing two groups of data. The statistical analysis software Prism version 9 was used. Unless otherwise indicated, the total number of samples is 16 (eight from Space and eight Earth samples).
