# Supplementary material for: Spaceflight alters molecular networks linked to diverse human diseases in a single cellular model
Source: Sci Adv. 2026 Jan 2;12(1):eadw7832. doi: 10.1126/sciadv.adw7832 (PMC12758520; doi:10.1126/sciadv.adw7832)
Supplement: Supplementary file 1 — Figs. S1 to S6 Table S1 Legends for data S1 to S10 [file sciadv.adw7832_sm.pdf]

Science Advances
AAAS

Supplementary Materials for

Spaceflight alters molecular networks linked to diverse human diseases in a single cellular model

Wijdan Al-Ahmadi *et al.*

Corresponding author: Khalid S. A. Khabar, khabar@kfshrc.edu.sa

Sci. Adv. **12**, eadw7832 (2026)
DOI: 10.1126/sciadv.adw7832

The PDF file includes:

Figs. S1 to S6
Table S1
Legends for data S1 to S10

Other Supplementary Material for this manuscript includes the following:

Data S1 to S10

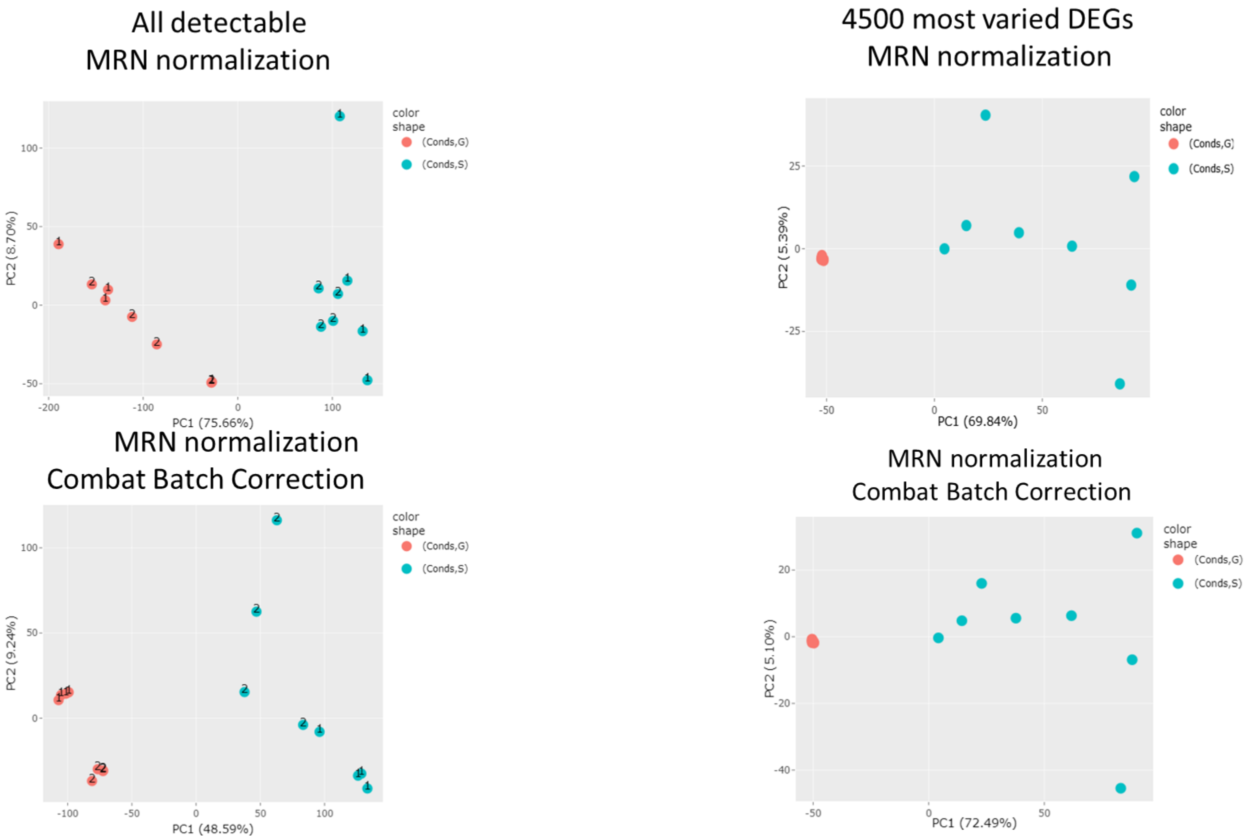

Fig. S1. Principal components analysis for the entire 16 samples from two batches (each of the two batches are four replicates) for Earth (G) vs Spaceflight.

.

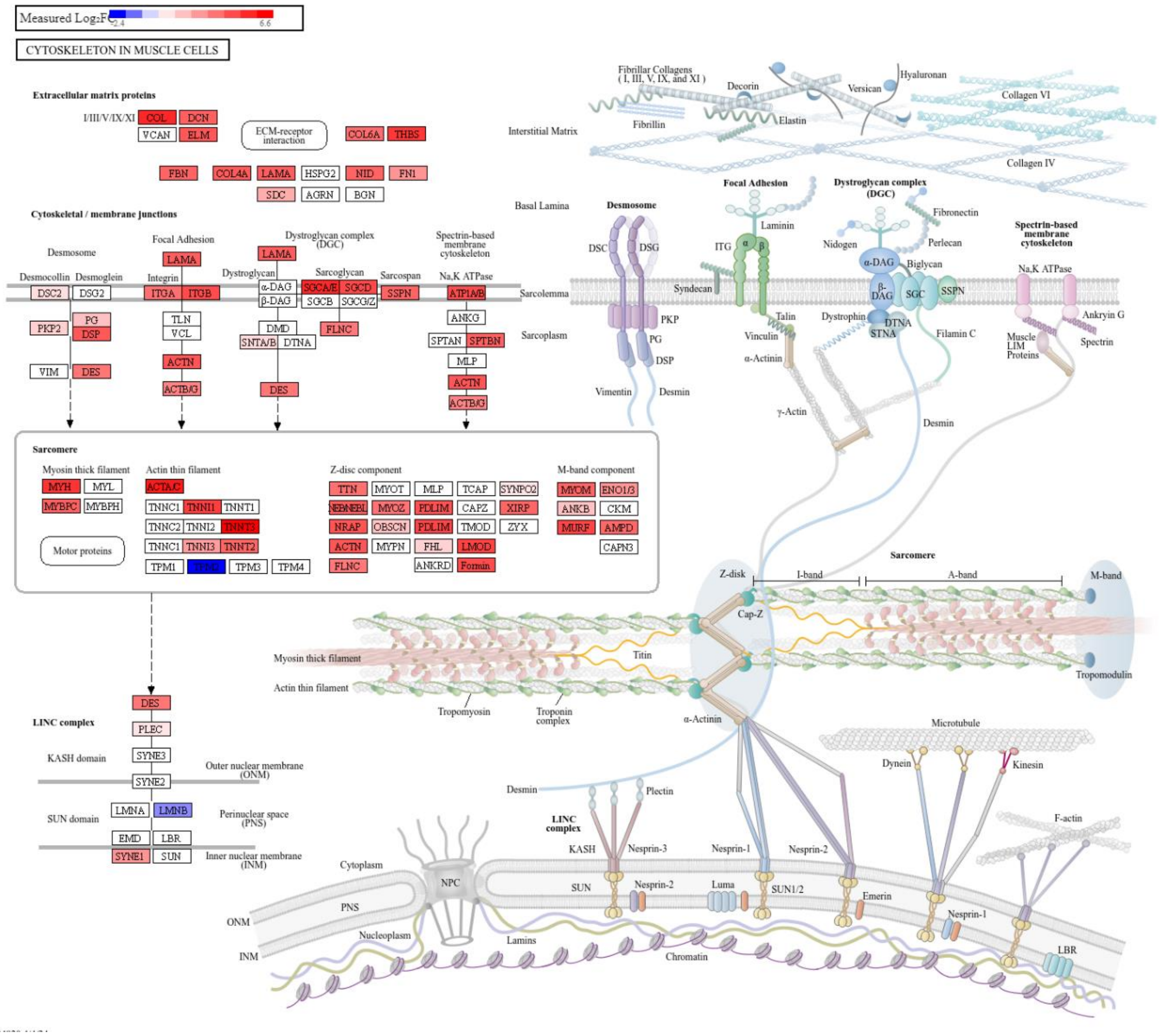

Fig. S2. Cytoskeleton in muscle cell. This web-generated KEGG pathway diagram overlayed with the computed perturbation of each gene. The perturbation accounts both for the gene's measured fold change and for the accumulated perturbation propagated from any upstream genes (accumulation). The highest negative perturbation is shown in blue, while the highest positive perturbation in red. The legend above describes the values on the gradient. Color intensity corresponds to the degree of change (legend is above).

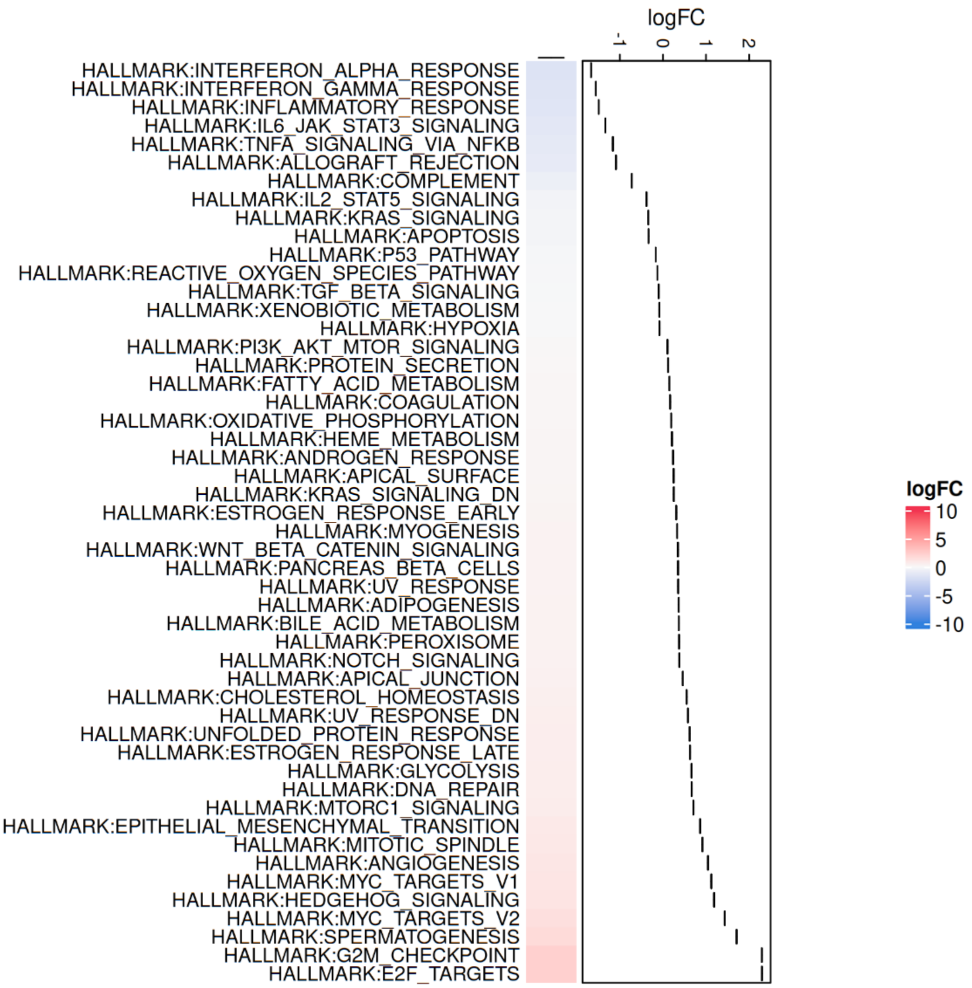

Fig. S3 Comparison between THP-1 cells and normal blood monocytes – enrichment clustering of MigDb Hallmarks. Enrichment analysis was performed with GSEA. Legend is color and intensity as shown.

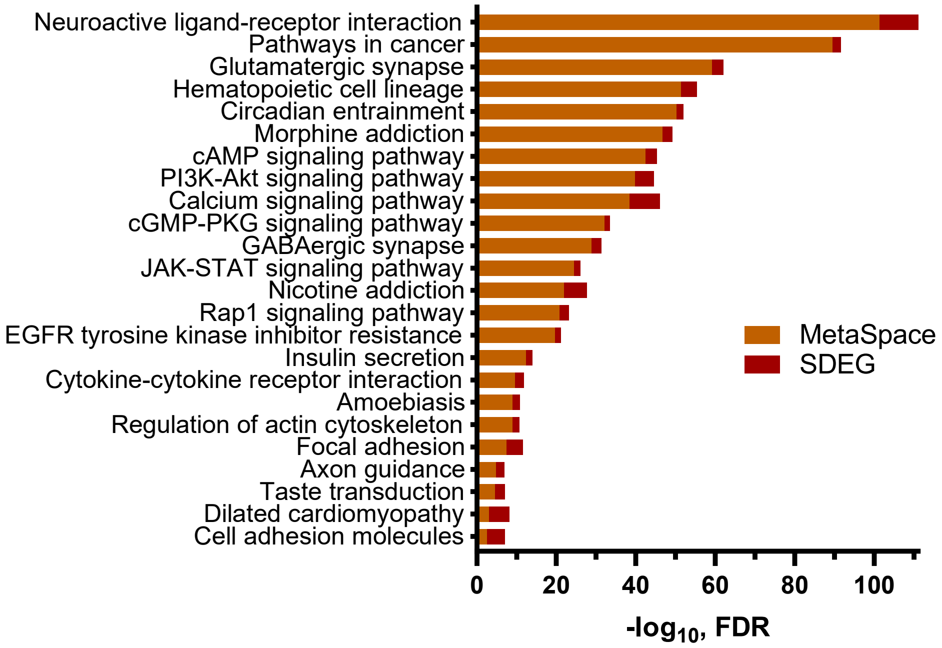

Fig. S4. Meta-analysis comparisons of common functional pathways between MetaMission Signature and SDEG. KEGG pathways were used to compare the 873 MetaSpace signature and our SDEG using MetaAnalysis ranking algorithm (IpathwayGuide software).

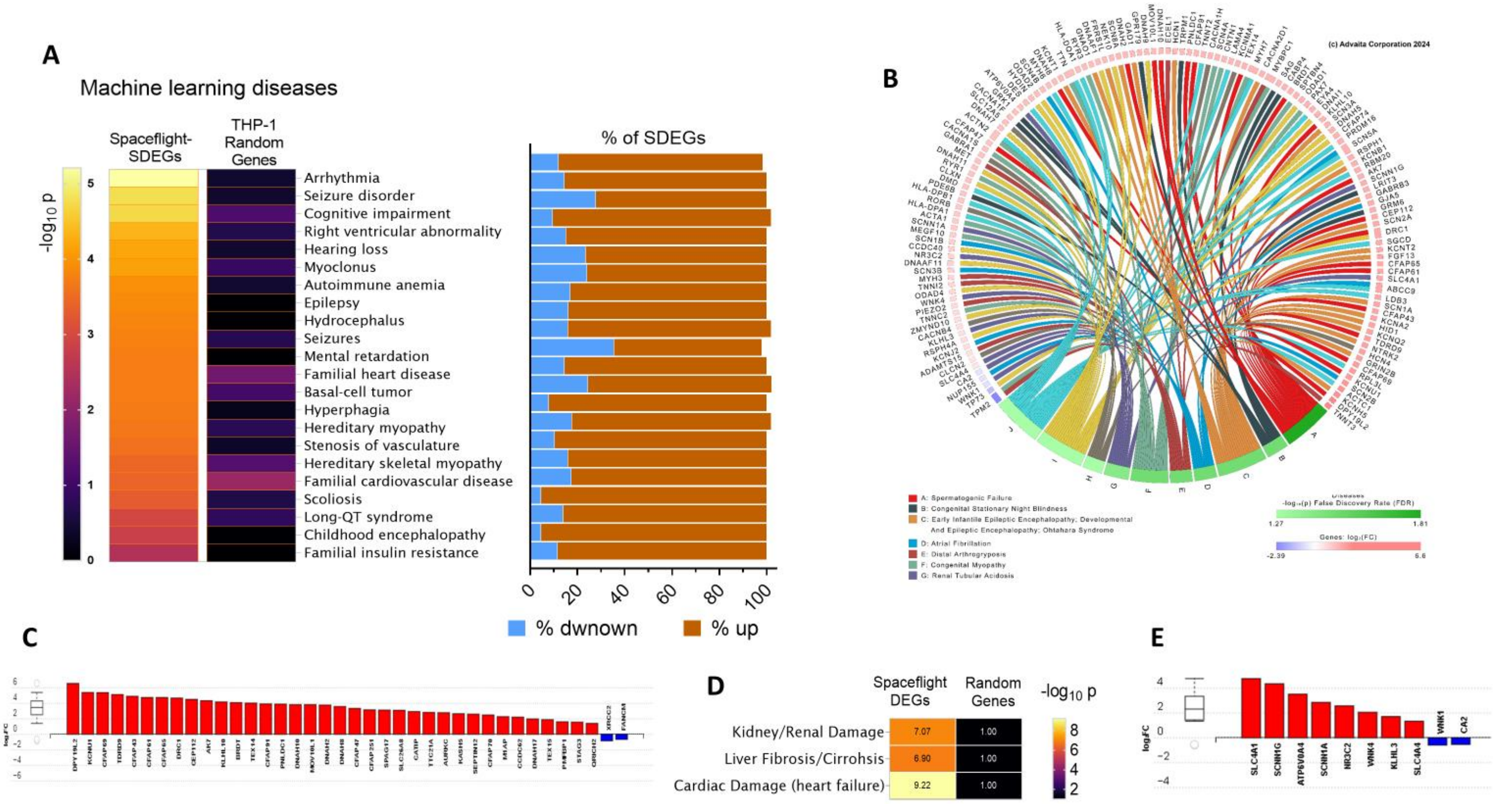

Fig. S5 Machine-learning health conditions/symptoms associated with spaceflight-altered cellular gene expression (A) Spaceflight SDEG and THP-1 random lists, each of 4522, were analyzed by IPA ML disease program. The list is ranked by p-value determined by Fisher's exact test. % genes in SDEGs that are either downregulated or upregulated are shown in the right panel. **(B)** Disease analysis was performed by the IPG program using an over-representation approach. For each disease, SDEGs were annotated to a disease term compared to the number of SDEGs in THP-1 background expected by chance. The p-value was computed using the hypergeometric distribution and corrected for multiple comparisons using FDR. The top significant disease annotations are shown (Green color legend). **(C)** Transcript measured expression $\log_2$ bar plot: SDEGs annotated to Spermatogenic failure. **D)** IPA ML disease program (Toxicological diseases database) was performed with both SDEGs and random THP-1 gene lists. The heatmap shows $-\log_{10}$ p-values (Fisher's Exact Test). **(E)** Transcript measured expression $\log_2$ bar plot of SDEGs annotated to Renal tubular acidosis and are ranked based on their $\log_2$ fold change. Upregulated genes are shown in red, and downregulated genes are shown in blue. The small boxes in C and E (left) depict the distribution of all the differentially expressed genes annotated to the indicated disease (Median, 1st, and 3rd quartiles).

D1: SFF18174

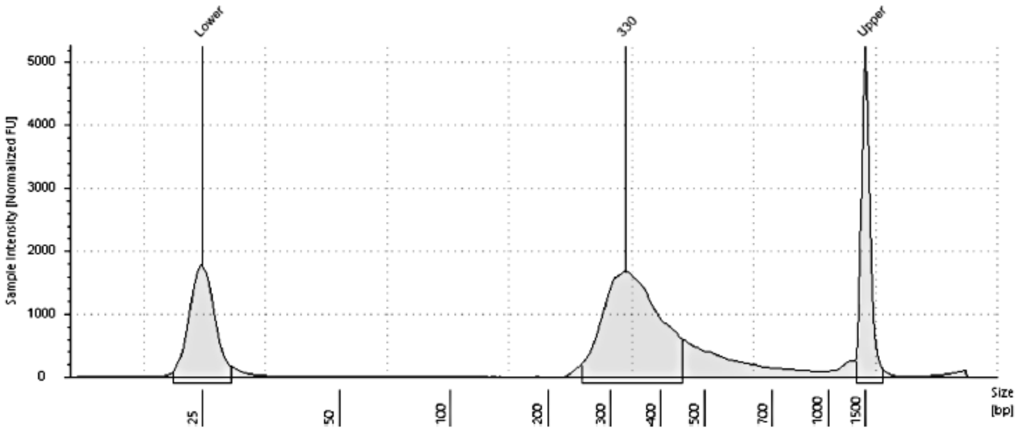

Sample Table

Well	Conc. [ng/µl]	Sample Description	Alert	Observations
D1	13.6	SFF18174		

Peak Table

Size [bp]	Calibrated Conc. [ng/µl]	Assigned Conc. [ng/µl]	Peak Molarity [nmol/l]	% Integrated Area	Peak Comment	Observations
25	6.51	-	401	-		Lower Marker
330	13.6	-	63.6	100.00		
1500	6.50	6.50	6.67	-		Upper Marker

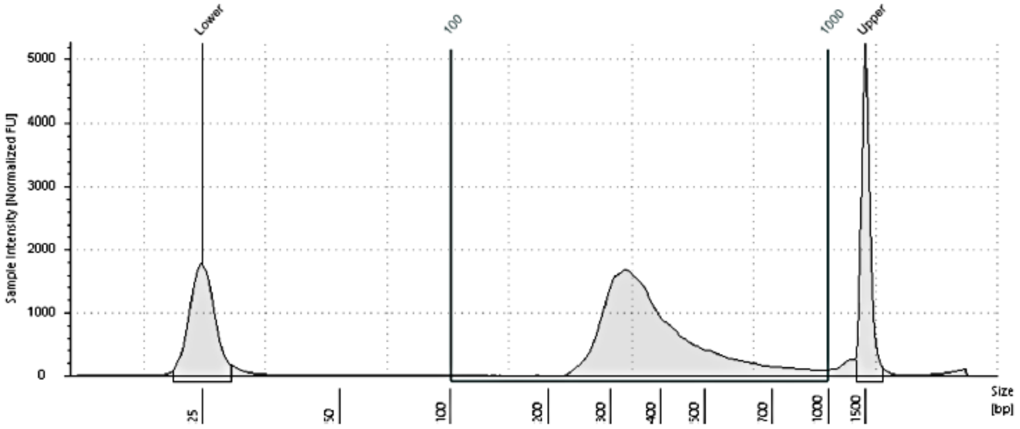

Region Table

From [bp]	To [bp]	Average Size [bp]	Conc. [ng/µl]	Region Molarity [nmol/l]	% of Total	Region Comment	Color
100	1000	405	18.1	74.2	93.06		

Fig. S6 Example of library size distribution

Table S1: Summary of top-enriched pathways and their scores

Pathway	ES or NES	FDR	SS	Database	METHOD
Extracellular matrix organization	2.52	1.7E-28	70.0	REACTOME	ORA_UP
Muscle contraction	2.68	5.8E-22	56.9	REACTOME	ORA_UP
Neuronal System	2.17	7.0E-23	48.0	REACTOME	ORA_UP
Diseases associated with O-glycosylation of proteins	2.87	1.9E-10	27.9	REACTOME	ORA_UP
Sensory Perception	2.09	5.4E-10	19.4	REACTOME	ORA_UP
Cell Cycle	2.80	1.3E-28	78.2	REACTOME	ORA_DN
mRNA Splicing - Major Pathway	3.17	1.7E-11	34.1	REACTOME	ORA_DN
DNA Repair	2.64	2.7E-11	27.9	REACTOME	ORA_DN
Chromatin organization	2.82	1.6E-10	27.6	REACTOME	ORA_DN
Cellular Senescence	3.08	2.6E-09	26.4	REACTOME	ORA_DN
Neuroactive ligand-receptor interaction	2.41	9.1E-20	45.9	KEGG	ORA_UP
Protein digestion and absorption	3.00	1.4E-15	44.5	KEGG	ORA_UP
Calcium signaling pathway	1.99	8.0E-11	20.1	KEGG	ORA_UP
ECM-receptor interaction	2.59	1.9E-10	25.2	KEGG	ORA_UP
Cell adhesion molecules	2.21	4.2E-10	20.8	KEGG	ORA_UP
DNA replication	7.63	8.9E-12	84.3	KEGG	ORA_DN
Base excision repair	5.00	1.5E-06	29.0	KEGG	ORA_DN
Cell cycle	3.25	1.7E-08	25.3	KEGG	ORA_DN
Systemic lupus erythematosus	3.51	1.1E-06	20.9	KEGG	ORA_DN
Neutrophil extracellular trap formation	3.04	1.5E-07	20.8	KEGG	ORA_DN
muscle differentiation	2.29	8.1E-15	32.3	HALLMARKS	ORA_UP
epithelial mesenchymal transition	2.18	5.4E-13	26.8	HALLMARKS	ORA_UP
"KRAS signaling, downregulated genes"	2.31	4.3E-12	26.3	HALLMARKS	ORA_UP
blood coagulation cascade	1.91	2.3E-05	8.9	HALLMARKS	ORA_UP
inflammation	1.71	2.3E-05	8.0	HALLMARKS	ORA_UP
cell cycle progression: G2/M checkpoint	4.88	2.2E-34	164.1	HALLMARKS	ORA_DN
cell cycle progression: E2F targets	4.79	1.6E-33	157.1	HALLMARKS	ORA_DN
"MYC targets, variant 1"	2.92	9.8E-11	29.2	HALLMARKS	ORA_DN
cell cycle progression: mitotic spindle assembly	2.64	3.2E-08	19.8	HALLMARKS	ORA_DN

Processing of Capped Intron-Containing Pre-mRNA	-3.64	2.2E-16	57.0	REACTOME	GSEA
Cell Cycle	-3.37	2.2E-16	52.8	REACTOME	GSEA
Chromatin modifying enzymes	-3.07	2.2E-16	48.1	REACTOME	GSEA
DNA Repair	-3.05	2.2E-16	47.8	REACTOME	GSEA
tRNA processing	-2.42	2.2E-16	37.9	REACTOME	GSEA
Extracellular matrix organization	2.27	2.2E-16	35.5	REACTOME	GSEA
Muscle contraction	2.02	3.5E-04	7.0	REACTOME	GSEA
Neuronal System	1.99	3.2E-04	6.9	REACTOME	GSEA
Sensory Perception	1.96	3.9E-04	6.7	REACTOME	GSEA
Immunoregulatory interactions between Lymphoid & a non-Lymphoid cell	1.89	5.0E-04	6.3	REACTOME	GSEA
Neuroactive ligand-receptor interaction	2.31	2.2E-16	36.2	KEGG	GSEA
Cell adhesion molecules	2.06	5.0E-04	6.8	KEGG	GSEA
Cytokine-cytokine receptor interaction	2.03	5.2E-04	6.6	KEGG	GSEA
Coronavirus disease	1.66	1.5E-02	3.0	KEGG	GSEA
Axon guidance	1.64	1.7E-02	2.9	KEGG	GSEA
Spliceosome	-3.42	2.2E-16	43.4	KEGG	GSEA
Nucleocytoplasmic transport	-2.92	2.2E-16	45.7	KEGG	GSEA
ATP-dependent chromatin remodeling	-2.77	2.2E-16	43.4	KEGG	GSEA
Cell cycle	-2.74	2.2E-16	43.0	KEGG	GSEA
Ribosome biogenesis in eukaryotes	-2.62	2.2E-16	43.0	KEGG	GSEA
KRAS_SIGNALING_DN	2.11	2.2E-16	43.0	HALLMARKS	GSEA
MYOGENESIS	2.01	2.2E-16	43.0	HALLMARKS	GSEA
HYPOXIA	1.90	1.7E-04	43.0	HALLMARKS	GSEA
EPITHELIAL_MESENCHYMAL_TRANSITION	1.92	2.1E-04	43.0	HALLMARKS	GSEA
INFLAMMATORY_RESPONSE	1.66	6.6E-03	43.0	HALLMARKS	GSEA
E2F_TARGETS	-3.81	2.2E-16	43.0	HALLMARKS	GSEA
G2M_CHECKPOINT	-3.65	2.2E-16	43.0	HALLMARKS	GSEA
MYC_TARGETS_V1	-2.95	2.2E-16	43.0	HALLMARKS	GSEA
DNA_REPAIR	-2.32	2.2E-16	43.0	HALLMARKS	GSEA
OXIDATIVE_PHOSPHORYLATION	-1.96	2.2E-16	43.0	HALLMARKS	GSEA

ORA: over-representation-based analysis for upregulated or downregulated SDEGs
GSEA: Gene Set Enrichment Analysis
ES: enrichment score (ORA), NES: normalized enrichment score (GSEA)
SS=significance score: (Absolute value ES/NES x -log10FDR)

Data S1. (Separate file)
DESEQ2 scores

Data S2. (Separate file)
Detailed Pathways Enrichments

Data S3. (Separate file)
GSEA ANALYSIS14KDEGs

Data S4. (Separate file)
Cell Type

Data S5. (Separate file)
E2F Targets Meta Mission Comparison

Data S6. (Separate file)
Sample Sequence reads stats

Data S7. (Separate file)
Mapping statistics

Data S8. (Separate file)
Background and Random Lists

Data S9. (Separate file)
Extracted external data

Data S10. (Separate file)
MetaMission & MetaSignature Lists
